# Higher physical activity levels mitigate synaptic protein loss and cognitive deterioration in aging and in Alzheimer’s disease: a 10-year longitudinal study

**DOI:** 10.1186/s43556-026-00513-5

**Published:** 2026-07-15

**Authors:** Shuiyue Quan, Xiaofeng Fu, Huimin Cai, Weiyun Zhang, Yumei Geng, Qing Tian, Ziye Ren, Yinghao Xu, Chengyu An, Jiaqi Li, Wei Wang, Longfei Jia

**Affiliations:** 1 Department of Neurology and Innovation Center for Neurological Disorders, National Clinical Research Center for Geriatric Diseases, Xuanwu Hospital, Capital Medical University, 45 Changchun St., Beijing, 100053 China; 2 Beijing Key Lab of Clinical Translational Research in Cognitive, Affective and Behavioral Disorders, Xuanwu Hospital, Capital Medical University, Beijing, China

**Keywords:** Alzheimer’s disease, Synaptic protein, Physical activity, Aging, Longitudinal

## Abstract

Synaptic degeneration is a hallmark of Alzheimer’s disease (AD) and is closely linked to cognitive decline. Although physical activity (PA) can preserve synaptic integrity and cognitive function, its long-term effects during aging and AD progression remain poorly characterized. Here, we conducted a 10-year longitudinal study to investigate the effects of different PA levels on synaptic proteins and cognitive function during aging and in AD progression. The study included 231 cognitively normal older adults (preclinical AD, 116; controls, 115), who were stratified based on their PA intensity. Plasma was collected every 2 years to quantify four synaptic proteins (GAP43, neurogranin, SNAP25, and synaptotagmin 1) in neuron-derived extracellular vesicles (EVs), with concurrent cognitive assessments. The results indicated that all synaptic proteins declined over time, with a greater reduction in AD than in normal aging (*P* < 0.05). Synaptic protein levels did not differ between PA groups at baseline (*P* > 0.05), but at the 10-year follow-up, participants with higher PA had significantly greater synaptic protein levels than those with lower PA in both AD and controls (*P* < 0.05). Consistently, higher PA alleviated synaptic protein loss during aging (*P* < 0.05). Furthermore, higher PA was associated with slower cognitive decline in patients with preclinical AD (*P* < 0.05). Our study suggests that higher levels of PA may mitigate age- and AD-related synaptic deterioration, thereby contributing to cognitive resilience in late life.

**Supplementary Information:**

The online version contains supplementary material available at 10.1186/s43556-026-00513-5.

## Introduction

The increasing incidence of Alzheimer’s disease (AD) has exacerbated the global health burden [1, 2]. However, effective treatments for AD are lacking, mainly because AD typically undergoes a prolonged asymptomatic period before clinical diagnosis, during which relatively irreversible pathological changes occur in the brain [3, 4]. Evidence indicates that synaptic damage is an early AD hallmark and is strongly correlated with cognitive impairment [5]. Therefore, elucidating the pattern of synaptic damage during this “silent period” and initiating early interventions may help delay or prevent symptom onset [6].

Unfortunately, owing to the difficulty in visualizing synaptic degeneration in neuropathological studies [7], dynamically quantifying synaptic loss in living patients is challenging. In contrast, neuron-derived extracellular vesicles (EVs) can carry synaptic proteins in the brain into the peripheral blood, where they can be noninvasively detected [8]. We previously demonstrated that the levels of several synaptic proteins in plasma neuron-derived EVs, including growth-associated protein 43 (GAP43), neurogranin (Ng), synaptosomal-associated protein 25 (SNAP25), and synaptotagmin 1, are reduced during the preclinical AD stage [4]. Other studies also found significantly lower synaptic protein levels in neuron-derived EVs of AD patients compared to controls [9, 10]. Collectively, these suggest that EVs are reliable biomarkers of synaptic damage in the AD brain. However, most studies were cross-sectional in design, providing only stage-specific information. Therefore, if monitored longitudinally at multiple time points [11], synaptic proteins in EVs may help comprehensively discern their relationship with AD progression and serve as sensitive indicators for evaluating intervention efficacy.

Synapses are modifiable targets of physical activity (PA) [12], the intensity of which is typically standardized using metabolic equivalents (METs) [13]. Evidence from animal and human studies indicates that PA can support synaptic homeostasis through various neurobiological mechanisms, including the upregulated brain-derived neurotrophic factors (BDNF), improved cerebral perfusion, reduced neuroinflammation, and enhanced synaptic plasticity [14, 15]. Additionally, regular exercise promotes synaptogenesis and increases neurotransmitter release, particularly in AD-vulnerable brain regions such as the hippocampus and entorhinal cortex [16]. Therefore, the synaptic effects of PA are believed to underlie its cognitive benefits [17]. In this regard, a large-scale prospective cohort study has shown that moderate PA levels are significantly associated with reduced dementia risk [18]. Even in autosomal dominant forms of dementia, higher PA levels have been closely linked to delayed symptom onset [19]. However, most studies linking PA to synaptic function included individuals already experiencing cognitive impairment. Consequently, the long-term effects of PA on synaptic and cognitive functions during the asymptomatic phase of AD remain largely unexplored. Moreover, limited studies have employed quantitative metrics of PA, such as METs, to longitudinally evaluate their relationships with synaptic protein alterations. In particular, it remains unclear whether higher PA levels provide additional synaptic benefits compared to lower levels. Hence, multi-timepoint longitudinal studies are necessary to dynamically track changes in synaptic biomarkers and cognitive performance during the preclinical AD stage under different PA levels, to explore their modifiability as intervention targets and elucidate the underlying biological mechanisms.

AD primarily affects adults over 65 and is closely linked to aging. During normal aging, the number of synaptic connections between neurons gradually declines [20], with mild but widespread reductions in synaptic protein expression [21]. These age-related synaptic changes are at least partially reversible [22] and may be improved through PA. Accordingly, animal studies confirm that early PA confers long-lasting protection against age-associated structural and functional synaptic impairment with region-specific effects [14, 23]. However, preclinical AD individuals have already experienced the accumulation of amyloid-β (Aβ) plaques and hyperphosphorylated tau [3], with synaptic injury likely being more progressive and disease-specific. Therefore, the regulatory effects of PA intensity on synaptic function may differ between healthy cognitive aging and the AD continuum. Consequently, it is necessary to evaluate longitudinal changes in synaptic proteins in cognitively intact older adults and determine whether the effect of PA on synaptic homeostasis depends on underlying AD pathology.

This study hypothesized that higher PA levels are linked to preserved synaptic proteins and cognitive function during aging. To evaluate this, we conducted a prospective cohort study examining 10-year longitudinal alterations of four synaptic proteins (GAP43, Ng, SNAP25, and synaptotagmin-1) in plasma neuron-derived EVs, focusing on the impact of PA levels as measured by METs. Specifically, this study aimed to evaluate the longitudinal trajectory of synaptic proteins in aging and AD progression, and investigate whether higher PA levels confer greater benefits on synaptic proteins and cognitive function during aging or the preclinical AD stage. Our findings provide the first longitudinal evidence for the modifiability of synaptic impairment, and support the development of targeted early intervention strategies.

## Results

### Participant characteristics

**Fig. 1:**
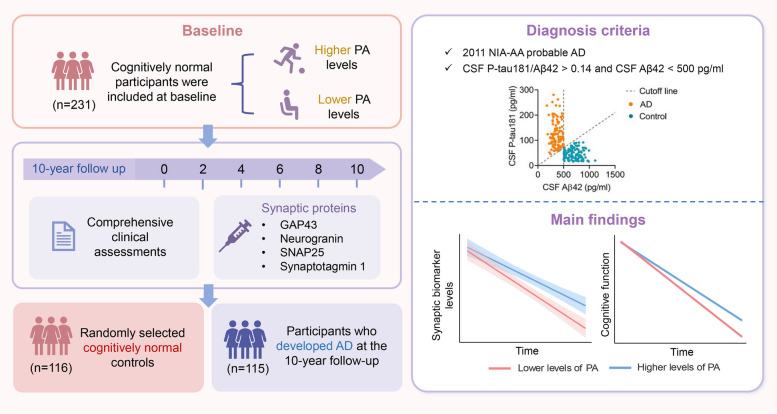
Schematic overview of the study design. This study was based on a 10-year nested case–control design within a longitudinal cohort initiated in 2014, including cognitively normal older adults at baseline. The thresholds of CSF P-tau181/Aβ42 ratio (0.14) and Aβ42 (500 pg/ml) were used to distinguish participants with preclinical AD from cognitively normal individuals. PA levels were assessed at all follow-up points, and patients were stratified into higher- and lower-PA groups based on METs. Plasma was collected every two years for neuron-derived EV isolation and quantification of synaptic biomarkers (GAP43, neurogranin, SNAP25, and synaptotagmin 1), along with comprehensive clinical assessments. CSF, cerebrospinal fluid; P-tau, phosphorylated tau; Aβ, amyloid-β; AD, Alzheimer’s disease; PA, physical activity; METs, metabolic equivalent thresholds; EVs, extracellular vesicles; GAP43, growth-associated protein 43; SNAP25, synaptosomal-associated protein 25

The overall study is illustrated in Fig. 1. Demographic and clinical information for all participants is summarized in Table 1. We blindly analyzed the plasma and cerebrospinal fluid (CSF) samples collected from 231 participants. No significant differences were observed between the AD and control groups in terms of age, sex, or educational level (*P* > 0.05). However, as expected, a significant difference in the percentage of *APOE* ε4 carriers was detected between the two groups (*P* < 0.05). Although Mini-Mental State Examination (MMSE) and Montreal Cognitive Assessment (MoCA) scores showed no differences at baseline (*P* > 0.05), significant differences were observed at the 10-year follow-up (*P* < 0.05). Compared with controls, patients with AD exhibited lower Aβ42 levels and higher T-tau and P-tau181 levels in CSF at the 10-year follow-up (*P* < 0.05).

**Table 1:** Characteristics of participants

		Baseline				At the 10-year follow-up			
Characteristic	Total Sample (*n* = 231)	Con-L (*n* = 58)	Con-H (*n* = 58)	AD-L (*n* = 60)	AD-H (*n* = 55)	Con-L (*n* = 58)	Con-H (*n* = 58)	AD-L (*n* = 60)	AD-H (*n* = 55)
Age, mean (SD), year	62.3 (6.8)	63.5 (6.9)	62.2 (6.4)	61.1 (6.3)	62.4 (7.5)	73.5 (6.9)	72.2 (6.4)	71.1 (6.3)	72.4 (7.5)
Educational attainment, mean (SD), year	9.2 (2.0)	9.3 (2.0)	9.2 (2.2)	9.4 (1.9)	8.9 (2.1)	9.3 (2.0)	9.2 (2.2)	9.4 (1.9)	8.9 (2.1)
Women, No. (%)	116 (50.2)	29 (50.0)	29 (50.0)	30 (50.0)	28 (50.9)	29 (50.0)	29 (50.0)	30 (50.0)	28 (50.9)
*APOE ε4* status, No. (% positive)	68 (29.4)	10 (17.2)	10 (17.2)	25 (41.7)	23 (41.8)	10 (17.2)	10 (17.2)	25 (41.7)	23 (41.8)
MMSE score, mean (SD)	27.2 (4.4)	30.0 (0)	30.0 (0)	29.8 (0.4)	29.8 (0.4)	28.9 (0.5)	29.0 (0.5)	19.0 (2.5)	21.0 (1.6)*
MoCA score, mean (SD)	25.4 (7.8)	30.0 (0)	30.0 (0)	28.7 (0.8)	28.2 (0.7)	28.3 (0.6)	28.3 (0.7)	13.4 (3.1)	16.2 (3.7)*
CSF biomarkers, mean (SD), pg/ml									
Aβ42, pg/ml	530.5 (190.5)	-	-	-	-	637.8 (101.4)	732.3 (149.3)*	361.1 (79.7)	389.1 (70.3)*
Aβ42/40	0.12 (0.07)	-	-	-	-	0.17 (0.07)	0.18 (0.06)	0.06 (0.01)	0.06 (0.01)
T-tau, pg/ml	500.2 (229.0)	-	-	-	-	362.4 (91.7)	317.8 (82.5)*	748.1 (201.2)	567.6 (184.2)*
P-tau181, pg/ml	86.1 (54.1)	-	-	-	-	47.4 (21.2)	53.8 (24.3)	121.9 (53.2)	121.9 (53.4)

Values for age, educational attainment, MMSE scores, MoCA score, and CSF biomarkers are shown as mean (SD), and group differences were compared using independent-sample t-tests. Sex and *APOE ε4* status were compared using χ^2^ tests

*Abbreviations: AD* Alzheimer’s disease, *MET* metabolic equivalent threshold, *Con-L* lower MET levels in control, *Con-H* higher MET levels in control, *AD-L* lower MET levels in AD, *AD-H* higher MET levels in AD, *APOE* apolipoprotein E, *MMSE* Mini-Mental State Examination, *MoCA* Montreal Cognitive Assessment, *CSF* cerebrospinal fluid, *Aβ* amyloid-β, *T-tau* total tau, *P-tau* phosphorylated tau, *SD* standard deviation

^*^P < 0.05 for comparisons between high- and low-MET groups within the same diagnostic status at the 10-year follow-up

Participants were further stratified into high- and low-MET groups based on their PA levels. At the 10-year follow-up, individuals with higher METs showed higher CSF Aβ42 levels and lower T-tau levels compared to those with lower METs in both groups (*P* < 0.05). Additionally, patients with AD with higher METs were more likely to have better cognitive performance (*P* < 0.05). No such differences were found within the controls (*P* > 0.05).

### Synaptic proteins in neuron-derived EVs decline more rapidly in AD than in aging

**Fig. 2:**
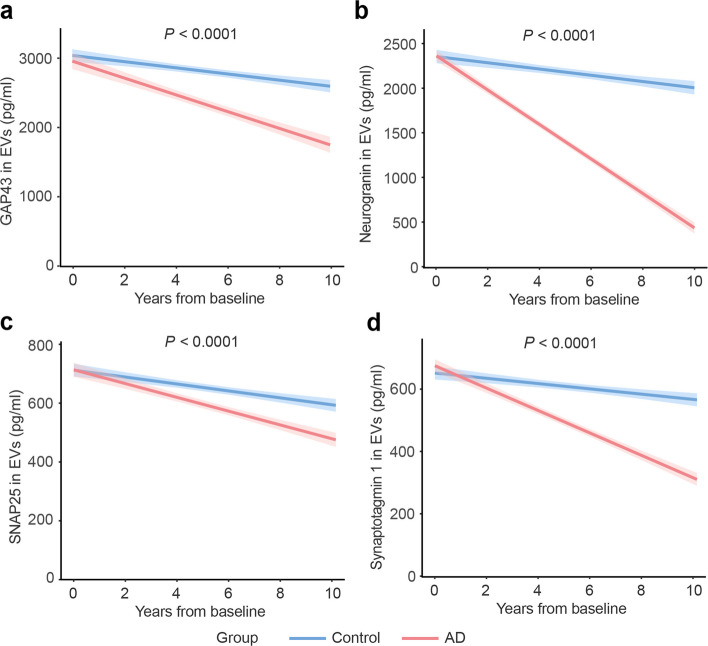
Longitudinal changes in synaptic proteins in neuron-derived extracellular vesicles. Longitudinal changes in growth-associated protein 43 (GAP43) (**a**), neurogranin (**b**), synaptosomal-associated protein 25 (SNAP25) (**c**), and synaptotagmin 1 (**d**) over a 10-year period in patients with preclinical AD and controls. The horizontal axis is the years elapsed from baseline. Shaded bands represent the 95% CIs for the regression slope, based on linear mixed-effects models incorporating the interaction between time and diagnosis. *n* = 116 (control), *n* = 115 (AD). AD, Alzheimer’s disease; CIs, confidence intervals

To characterize longitudinal patterns of synaptic protein changes during aging and AD progression, we analyzed the levels of GAP43, Ng, SNAP25, and synaptotagmin 1 in neuron-derived EVs collected at 0, 2, 4, 6, 8, and 10 years of follow-up. We first compared synaptic protein levels between the two groups at baseline and at the end of follow-up. Consistent with our previous findings [4], synaptic protein levels were significantly lower in patients with AD than in controls at the 10-year follow-up (*P* < 0.05; Fig. S1), although no significant differences were detected between the two groups at baseline (*P* > 0.05; Fig. S2). Furthermore, we described within-group longitudinal changes during aging and AD. Results showed that each synaptic protein was significantly altered during the 10-year follow-up period in both groups, exhibiting a generally progressive decline over time (*P* < 0.05; Table S1), indicating their participation in age- and AD-related pathological progression. Therefore, we performed linear mixed-effects models with the interaction between follow-up time and diagnostic status as an independent variable, to determine whether the rate of decline differed between groups. Our analysis revealed that all synaptic proteins exhibited a more pronounced longitudinal decline in patients with AD than in controls (*P* < 0.05; Fig. 2; Table S2), further supporting previous findings regarding their potential as AD-specific biomarkers [4].

### Differences in synaptic protein levels in neuron-derived EVs between high and low MET groups

**Fig. 3:**
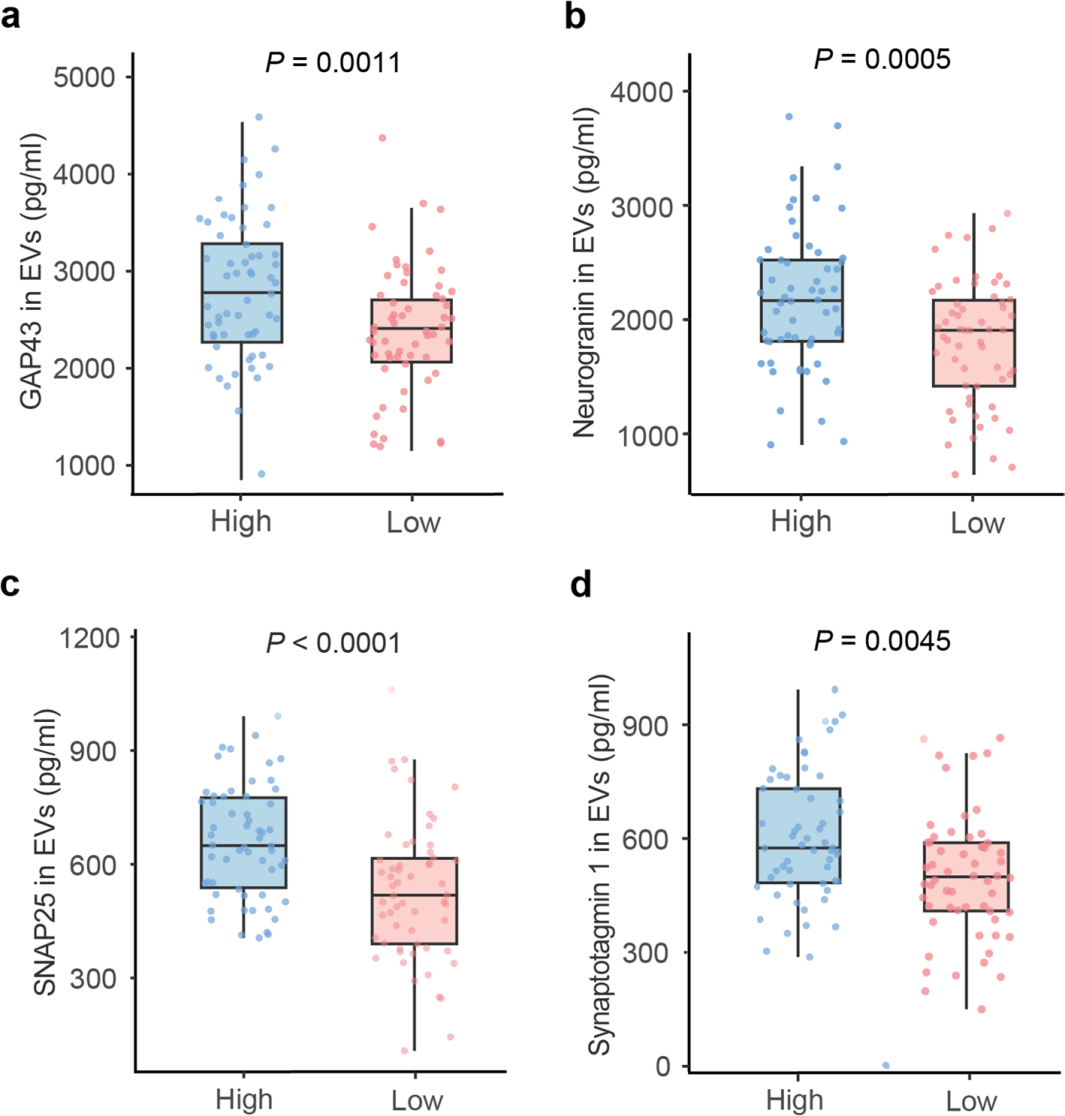
Neuron-derived EV levels of synaptic proteins at the 10-year follow-up in controls. Neuron-derived EV levels of growth-associated protein 43 (GAP43) (**a**), neurogranin (**b**), synaptosomal-associated protein 25 (SNAP25) (**c**), and synaptotagmin 1 (**d**) were measured at the 10-year follow-up in controls. *P* values obtained from t tests comparing high- versus low-MET groups are shown in each respective panel. *n* = 58 (low METs), *n* = 58 (high METs). EV, extracellular vesicle; MET, metabolic equivalent threshold

**Fig. 4:**
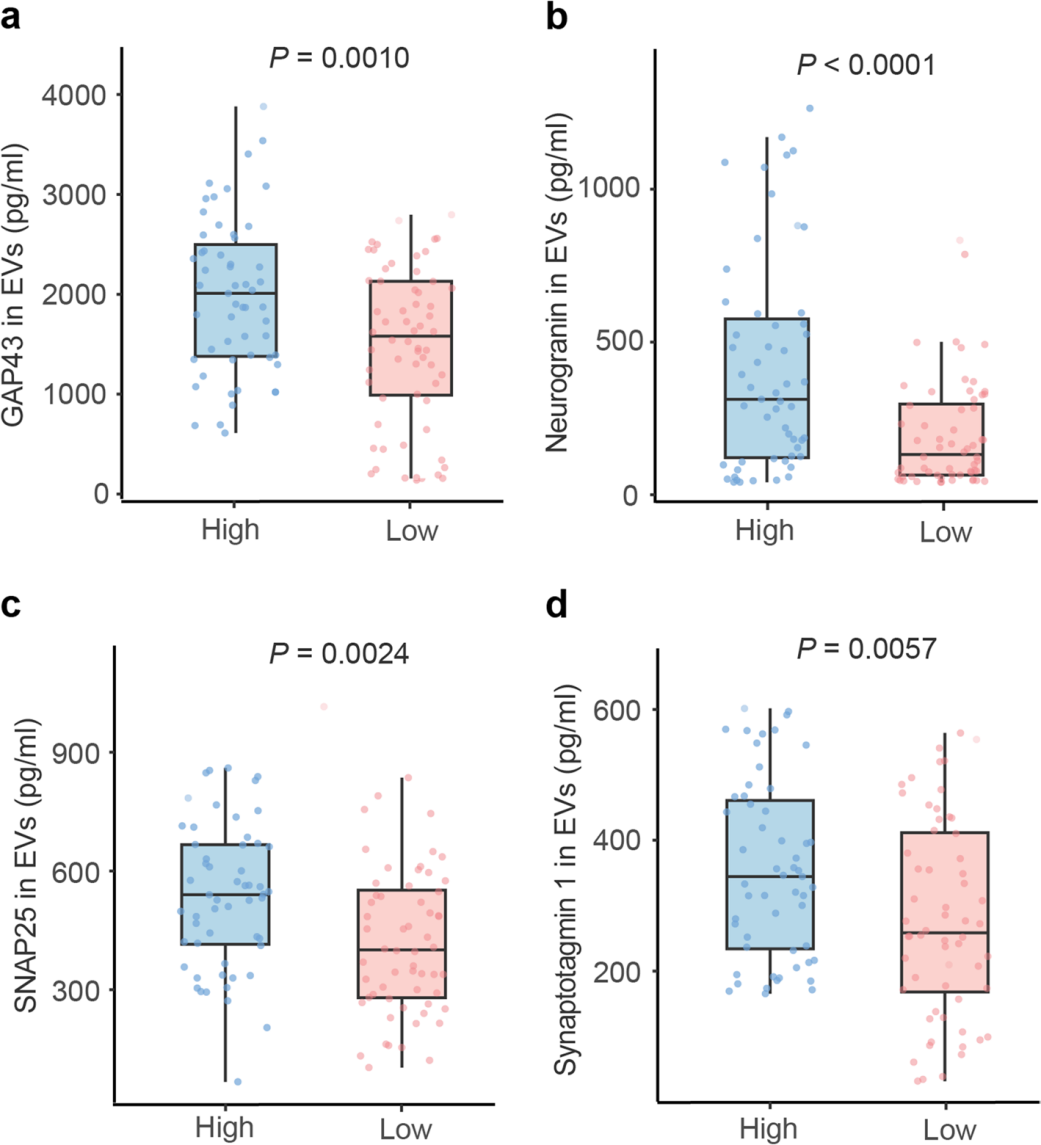
Neuron-derived EV levels of synaptic proteins at the 10-year follow-up in patients with Alzheimer’s disease. Neuron-derived EV levels of growth-associated protein 43 (GAP43) (**a**), neurogranin (**b**), synaptosomal-associated protein 25 (SNAP25) (**c**), and synaptotagmin 1 (**d**) were measured at the 10-year follow-up in patients with AD. *P* values obtained from t tests comparing high- versus low-MET groups are shown in each respective panel. *n* = 60 (low METs), *n* = 55 (high METs). EV, extracellular vesicle; MET, metabolic equivalent threshold; AD, Alzheimer’s disease

To evaluate whether PA intensity influences synaptic protein concentrations, we first compared synaptic protein levels between the high- and low-MET groups at the 10-year follow-up visit. We observed that in both the AD and control groups, individuals with higher MET levels exhibited significantly higher levels of GAP43, Ng, SNAP25, and synaptotagmin 1 than those with lower MET levels (*P* < 0.05; Fig. 3 and 4). Then, we examined whether this difference in the levels of these synaptic proteins was already present at baseline, but found no significant differences between both groups (*P* > 0.05; Figs. S3 and S4). Together, these findings provide preliminary evidence that PA is associated with maintained synaptic protein stability and may be linked to certain neuroprotective effects during aging and AD progression.

### Higher physical activity levels are associated with reduced synaptic protein loss in neuron-derived EVs in controls

To further assess whether PA levels modified synaptic protein changes over time during aging, we performed within-group longitudinal analyses in the control group, stratified by MET levels. The results showed that GAP43, Ng, and synaptotagmin 1 levels declined over the 10-year follow-up in both the low- and high-MET groups (*P* < 0.05; Table S3). Notably, SNAP25 levels exhibited a significant decline only in the low-MET group (*P* < 0.05; Table S3), whereas no statistically significant change was observed in the high-MET group (*P* = 0.07; Table S3). These results suggest that PA may be associated with the preservation of synaptic integrity during aging, particularly of SNAP25, ultimately contributing to an enhanced cognitive reserve.

**Fig. 5:**
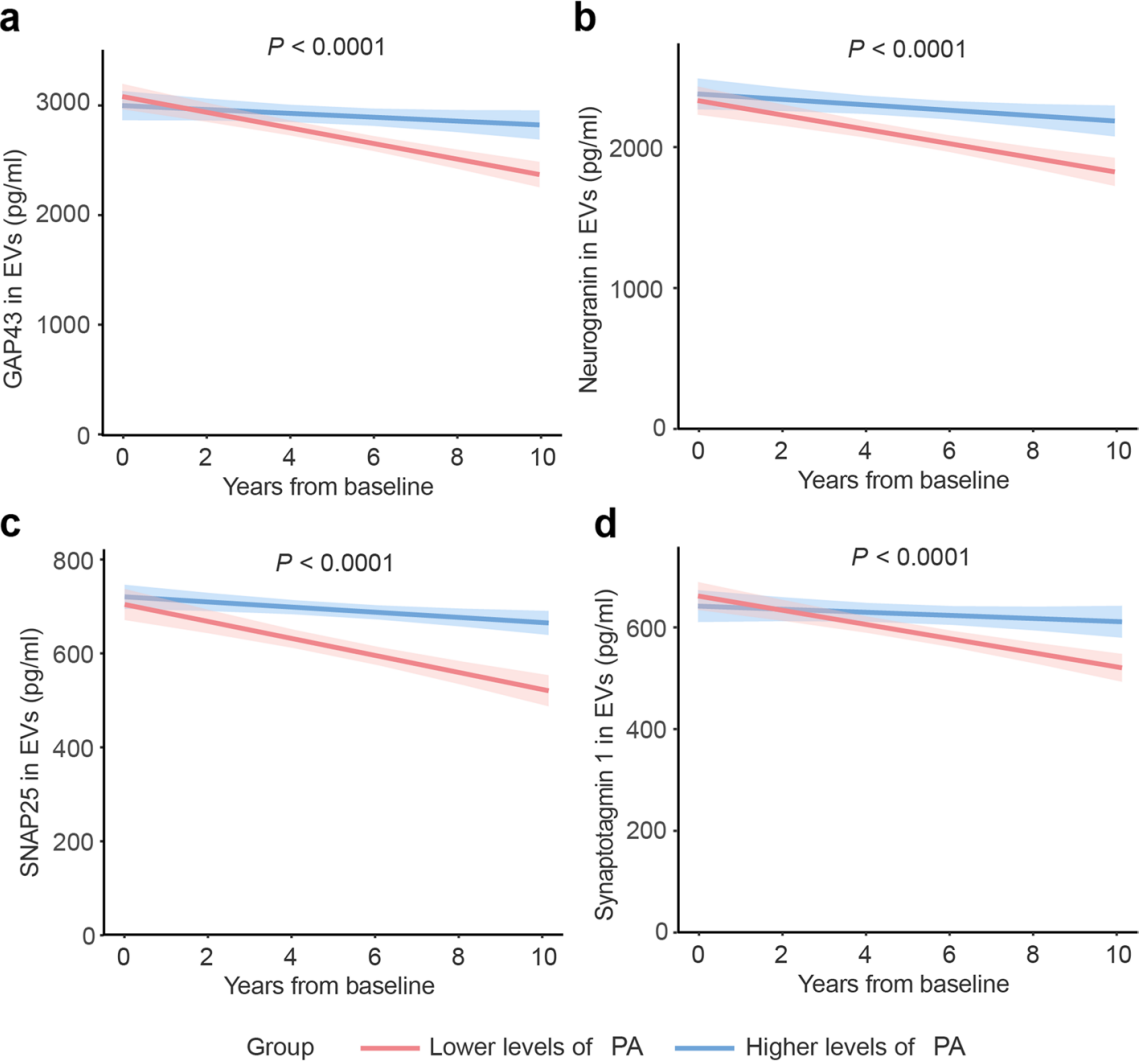
Longitudinal changes in synaptic proteins in neuron-derived EVs in controls by MET levels. Longitudinal changes in growth-associated protein 43 (GAP43) (**a**), neurogranin (**b**), synaptosomal-associated protein 25 (SNAP25) (**c**), and synaptotagmin 1 (**d**) were assessed over a 10-year follow-up period among cognitively normal older adults, who were stratified into high and low MET groups. The horizontal axis is the years elapsed from baseline. Shaded bands represent the 95% CIs for the regression slope, based on linear mixed-effects models incorporating the interaction between time and MET status. *n* = 58 (low METs), *n* = 58 (high METs). EVs, extracellular vesicles; MET, metabolic equivalent threshold; CIs, confidence intervals

Subsequently, we conducted linear mixed-effects model analyses using the interaction between follow-up time and PA level groups as the independent variable to further elucidate these differences. The levels of all four synaptic proteins declined steeply in the low-MET group (*P* < 0.05; Fig. 5; Table S2), further supporting the potential association between PA and mitigated age-related synaptic deterioration. To further delineate these longitudinal associations, we performed sensitivity analyses treating PA as a continuous variable using EV samples collected at 0, 2, 4, 6, 8, and 10 years of follow-up. The results showed patterns consistent with the primary analyses, with higher MET levels generally associated with relatively preserved synaptic protein levels at later follow-up visits (Fig. S5).

To explore the peripheral molecular mechanisms linking PA to synaptic preservation, we quantified four exerkines in plasma samples collected at the 10-year follow-up, including BDNF, cathepsin B (CTSB), insulin-like growth factor 1 (IGF-1), and irisin. The results showed that participants in the high-MET group exhibited significantly higher plasma concentrations of all four exerkines compared to the low-MET group (*P* < 0.05; Fig. S6).

Together, these results imply that higher PA levels were related to reduced synaptic protein loss during aging, with variability across synaptic proteins potentially reflecting differences in PA sensitivity.

### Higher physical activity levels are associated with attenuated synaptic protein loss in neuron-derived EVs in preclinical AD

To investigate how PA influences AD-related synaptic changes, we examined changes in synaptic proteins during the preclinical AD phase across the MET subgroups. As expected, group-wise longitudinal analyses showed all four synaptic proteins displayed decreasing trends over the 10-year follow-up period in the low- and high-MET groups, with the low-MET group exhibiting a significantly steeper decline (*P* < 0.05; Table S4). Notably, unlike the pattern observed in controls, SNAP25 levels also declined significantly in the high-MET group (*P* < 0.05; Table S4), implying that PA may be less effective at counteracting synaptic deterioration in the context of AD pathology.

**Fig. 6:**
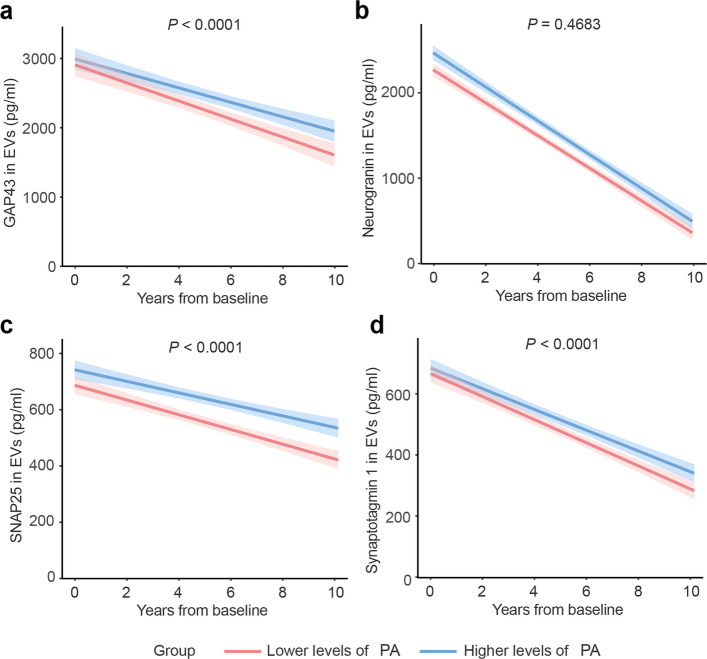
Longitudinal changes in synaptic proteins in neuron-derived EVs during Alzheimer’s disease progression by MET levels. Longitudinal changes in growth-associated protein 43 (GAP43) (**a**), neurogranin (**b**), synaptosomal-associated protein 25 (SNAP25) (**c**), and synaptotagmin 1 (**d**) were assessed over a 10-year follow-up period among patients with preclinical AD, who were stratified into high and low MET groups. The horizontal axis is the years elapsed from baseline. Shaded bands represent the 95% CIs for the regression slope, based on linear mixed-effects models incorporating the interaction between time and MET status. *n* = 60 (low METs), *n* = 55 (high METs). EVs, extracellular vesicles; MET, metabolic equivalent threshold; AD, Alzheimer’s disease; CIs, confidence intervals

Therefore, to explore whether PA exerts differential effects on synaptic biomarkers under pathological conditions, we analyzed synaptic protein trajectories across PA subgroups in patients with AD using the same approach as in the controls. The results indicated that GAP43, SNAP25, and synaptotagmin 1 levels declined more rapidly in the low-MET group (*P* < 0.05; Fig. 6; Table S2), whereas the rate of Ng decline was comparable between the two groups (*P* = 0.47; Fig. 6; Table S2). This divergence suggests that the link between PA and synaptic preservation may be limited by the underlying neuropathological burden, with synaptic responsiveness varying across pathological states. In addition, sensitivity analyses modeling PA as a continuous variable showed overall patterns consistent with these findings (Fig. S7). We similarly measured the four exerkines (BDNF, CTSB, IGF-1, and irisin) in plasma samples from preclinical AD participants at the 10-year follow-up and obtained results similar to those in the control group (all *P* < 0.05; Fig. S8).These findings imply that higher PA levels are associated with partial attenuation of synaptic protein loss during the preclinical AD stage, with the magnitude of these associations varying by synaptic protein type and underlying pathological context.

### Higher physical activity is associated with slower cognitive decline in preclinical AD

**Fig. 7:**
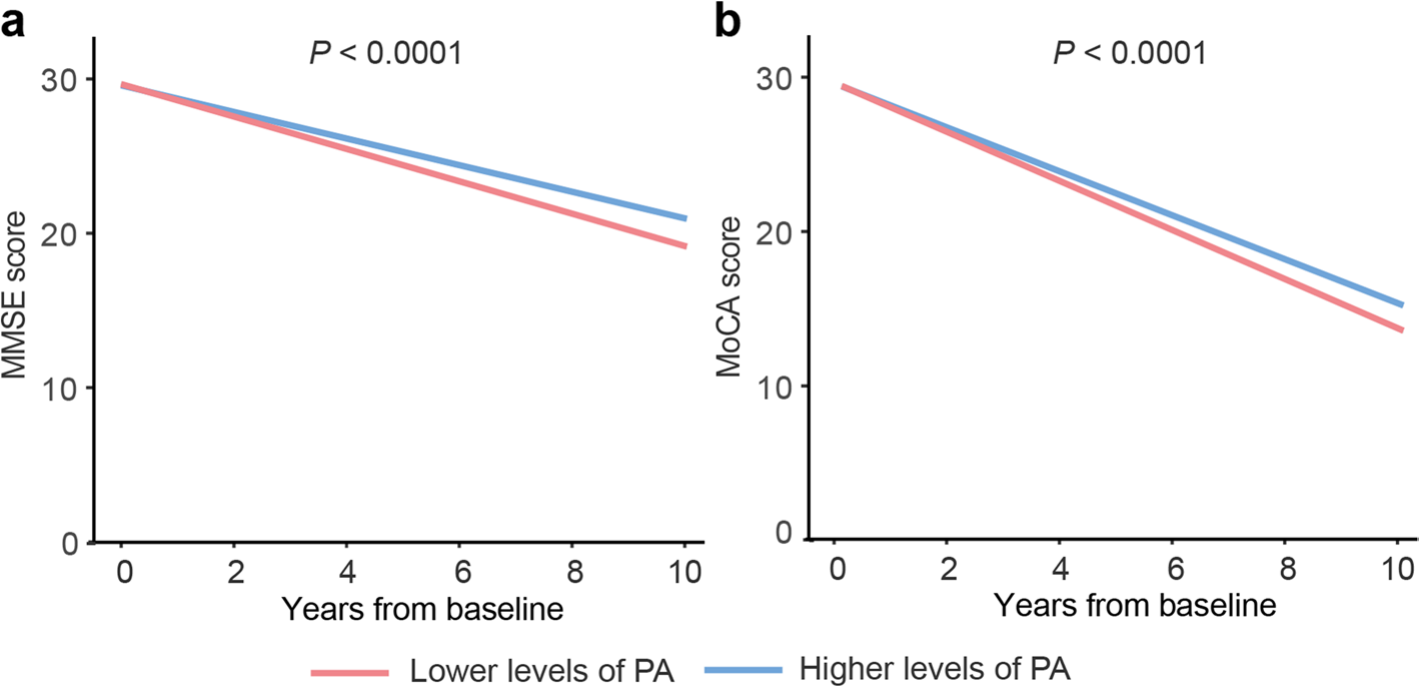
Longitudinal cognitive function changes in participants with preclinical Alzheimer’s disease by MET levels. Longitudinal changes in Mini-Mental State Examination (MMSE) (**a**) and Montreal Cognitive Assessment (MoCA) (**b**) scores over a 10-year follow-up period among patients with preclinical AD, who were stratified into high and low MET groups. The horizontal axis is the years elapsed from baseline. Shaded bands represent the 95% CIs for the regression slope, based on linear mixed-effects models incorporating the interaction between time and MET status. *n* = 60 (low METs), *n* = 55 (high METs). MET, metabolic equivalent threshold; AD, Alzheimer’s disease; CIs, confidence intervals

Given the observed synaptic benefits, we assessed whether the protective effects of PA extended to cognitive preservation in individuals with preclinical AD, as evaluated by MMSE and MoCA scores. Results from linear mixed-effects model analyses showed that participants with higher MET levels exhibited significantly slower cognitive decline than those with lower MET levels, as reflected in both MMSE and MoCA scores (*P* < 0.05; Fig. 7; Tables S2 and S4). The consistent patterns further support the notion that PA-associated synaptic resilience may functionally contribute to delayed cognitive deterioration in patients with preclinical AD.

## Discussion

In this study, we comprehensively examined the longitudinal changes in four synaptic biomarkers (including GAP43, Ng, SNAP25, and synaptotagmin 1), derived from neuronal EVs in the blood during aging and AD progression, with an emphasis on the effects of PA levels. Our findings suggested that each synaptic protein displayed a significant progressive reduction in both groups, with more pronounced decreases detected in the AD group. Notably, these declines appeared to be attenuated among participants with higher MET levels, indicating a modulatory role of PA in preserving synaptic integrity. To our knowledge, this is the first study to systematically track longitudinal changes in key synaptic proteins related to PA intensity across aging and the preclinical AD stage over a decade. Overall, our findings highlighted the greater buffering effect of higher PA levels on age-related and disease-specific synaptic deterioration, ultimately supporting cognitive health.

Tracking synaptic protein dynamics in preclinical AD is crucial, as these early synaptic alterations are closely associated with subsequent disease progression [24]. Therefore, we included cognitively normal older adults in this 10-year longitudinal cohort study, with those who developed AD during follow-up classified as having preclinical AD at baseline. We continuously monitored the levels of the four synaptic proteins in neuronal EVs and compared their longitudinal trajectories during aging and AD progression. Our results showed significant longitudinal declines in synaptic protein levels in the aging and AD groups, suggesting shared early synaptic deterioration and an intrinsic pathological connection between them. These results are consistent with a previous study wherein APP/PS1 transgenic mice displayed progressively extensive synaptic damage in an age-dependent manner [25]. Notably, the decline in synaptic protein levels was more pronounced in AD than in aging individuals. This implies that AD patients may be affected by more severe and specific molecular and cellular mechanisms, such as the well-established synaptotoxic Aβ and tau oligomers [12, 26]. Our results also support previous findings that normal aging leads to a relatively mild loss of synapses (approximately 10%–15%) [27], compared to that in AD brains (≥ 44% in certain regions) [28]. Thus, cognitively healthy aging may be attributed to compensatory neural mechanisms that collectively sustain synaptic homeostasis despite the presence of age-related stressors.

An increasing number of AD-related studies have identified synapses as modifiable therapeutic targets that can be effectively regulated by PA intensity. For instance, a postmortem cohort study demonstrated that individuals with higher late-life PA levels displayed elevated expression of multiple synaptic markers in brain tissue, including synaptophysin, vesicle-associated membrane protein, SNAP25, synaptotagmin-1, and syntaxin [29]. Correspondingly, findings from the Rush Memory and Aging Project confirmed that older adults with higher PA levels exhibited preserved cognitive function and enhanced synaptic integrity compared to their less active counterparts [30]. Preclinical studies using AD animal models have also indicated that exercise plays a pivotal role in enhancing synaptic function and facilitating cognitive improvement [31]. Notably, our current findings are fully consistent with this cumulative body of evidence. Specifically, linear mixed-effects model analysis revealed that higher PA levels correlated with elevated concentrations of several synaptic proteins in neuron-derived EVs, and slower rates of cognitive decline during the preclinical AD stage. These results imply that PA may boost cognitive reserve by promoting synaptic vesicle transport, neurotransmitter release, and synaptic plasticity. Mounting evidence further delineates that exercise mediates these benefits through diverse neurobiological pathways. In response to exercise, the body generates a range of “exerkines” that can cross the blood–brain barrier and exert direct or indirect effects on the brain [32]. For example, PA-induced lactate promotes the lactylation of synaptic proteins, such as synaptosome-associated protein 91 (SNAP91), thereby enhancing synaptic structure and cortical network function [33]. Consistently, “runner plasma” collected from voluntarily running mice and injected into sedentary mice significantly reduced neuroinflammation and improved cognition, an effect associated with the anti-inflammatory protein clusterin [34]. Other identified exerkines with potential neuromodulatory effects include BDNF, CTSB, IGF-1, and irisin, among others [35]. In this study, we observed corresponding upregulation of these exerkines (BDNF, CTSB, IGF-1, and irisin) in the higher PA groups, further supporting a mechanistic link between PA-mediated exerkine regulation and enhanced synaptic function, which may contribute to improved cognitive outcomes in at-risk older adults. Furthermore, PA confers complementary synaptoprotective and cognitive benefits by inhibiting aberrant microglial activation and remodeling the pro-inflammatory neural microenvironment [30]. Overall, further research focusing on these molecular pathways and expanding regulatory networks, particularly regarding the roles of exerkines in Aβ/tau metabolism and modulation of neuroinflammation, may facilitate the development of targeted interventions for future clinical trials in patients with AD.

Numerous studies have linked PA levels to successful cognitive aging. “Superagers”, who exhibit exceptional preservation of cognitive function with age, are typically more physically active than their age-matched counterparts [32]. Likewise, older adults who engage in PA at specific MET levels display better cognitive performance than sedentary individuals, with an inverted U-shaped relationship evident for PA intensity and cognitive outcomes [36]. However, the biological mechanisms underlying this beneficial association remain elusive, as age-related cognitive decline stems from diverse and interrelated pathological processes, particularly impaired synaptic plasticity and synaptic aging [37]. As a well-established core neurobiological basis for cognitive decline, synaptic aging triggers progressive synaptic dysfunction in the cerebral cortex and hippocampus with increasing age, manifested as impaired synaptic transmission efficacy, reduced synaptic contact density, synaptic mitochondrial dysfunction, and simplified dendritic arborization [6, 38, 39]. Consistently, a comprehensive synaptome atlas across the mouse lifespan has revealed age-dependent changes in synaptic density, morphology, and subtype composition, with widespread synaptic loss and dedifferentiation becoming particularly evident in older age [40]. In this regard, our population-based findings provide preliminary evidence that higher PA levels may confer critical synaptoprotective effects that mitigate age-related cognitive deterioration, suggesting that PA may represent a promising non-pharmacological strategy for delaying synaptic aging.

Notably, the synaptoprotective effects of PA varied markedly across distinct pathological conditions. During physiological aging, higher PA levels effectively mitigated declines in all four synaptic proteins assessed. In contrast, during the preclinical AD stage, this protective effect appeared partially constrained, most notably for Ng and SNAP25. These findings suggest that synaptic responsiveness to PA is not uniform across molecular components and becomes increasingly limited in the presence of AD-related pathology. This heterogeneity may partly reflect functional differences among synaptic proteins. SNAP25 primarily mediates presynaptic vesicle fusion and neurotransmitter release, whereas other proteins are more closely associated with synaptic plasticity or signaling processes [41]. This may render some proteins disproportionately vulnerable to AD-related pathological changes that disrupt synaptic homeostasis [12, 42]. Among them, Ng is a postsynaptic protein that regulates calmodulin availability and plays a critical role in long-term potentiation and synaptic plasticity. Emerging evidence indicates that Ng is vulnerable to disruption of its interaction with signaling partners by tau pathology, which promotes its leakage from synapses [43]. This vulnerability may partly explain why Ng shows diminished responsiveness to PA during the preclinical AD stage in our study. Nevertheless, this blunted responsiveness does not indicate that PA fails to counteract synaptic damage linked to AD-related pathology. In fact, PA attenuates the deleterious relationship between synaptic integrity and tau pathology, reinforcing its role as a buffer against tau-mediated synaptic deterioration, although no such association was observed with Aβ [12]. These findings indicate that the synaptoprotective effects of PA may vary dynamically with pathological status. Future studies are warranted to identify additional PA-responsive synaptic proteins and to clarify how their distinct biological functions contribute to the differential effects of exercise on synaptic health.

This study holds significant clinical application value and translational potential. First, we found that synaptic proteins in plasma neuron-derived EVs can be used for the dynamic monitoring of synaptic function [3, 44], overcoming the limitations of previous studies that relied on neuroimaging or postmortem brain tissue and failed to achieve longitudinal tracking in living individuals [12, 29]. This accessible biomarker enables routine non-invasive assessment of synaptic health in community-dwelling older adults, offering a novel tool for dynamically monitoring intervention effects in AD and other neurodegenerative disorders. Second, PA, as a low-cost and highly safe cognitive protection strategy, is suitable for long-term intervention among older adults, providing a simple and feasible non-pharmacological approach for the early prevention of AD.

Despite the positive results, our study has some limitations. First, although PA levels were assessed at each follow-up time point, they were evaluated using self-reported questionnaires rather than continuous 24-h objective monitoring, which limited our ability to precisely quantify cumulative PA intensity and duration over the follow-up period. In addition, questionnaire-based PA assessment is subject to recall bias or social desirability bias, which may lead participants to overestimate or underestimate their actual PA levels. Moreover, self-reported questionnaires are limited in capturing routine lifestyle activities (e.g., walking, stair climbing), potentially introducing measurement error and an underestimation of the true relationships between PA and synaptic outcomes. Nonetheless, our grouping method was designed to minimize the risk of misclassification, which is unlikely to have materially affected the primary conclusions of this study. Future studies are warranted to adopt objective and continuous PA monitoring tools (e.g., wearable accelerometers and fitness trackers) to collect real-time data on PA intensity, duration, and frequency, which will help to accurately explore the dose-dependent and cumulative effects of PA on synaptic plasticity and cognitive function in aging and preclinical AD [45, 46]. Second, neural cell adhesion molecule (NCAM)-based immunocapture was used to enrich neuron-derived EVs; however, due to the non-exclusive neuronal expression of NCAM and the heterogeneity of circulating EVs, non-neuronal contributions cannot be entirely excluded [47, 48]. Importantly, our enriched samples showed high expression of the L1 cell adhesion molecule (L1CAM), a specific marker for neuron-derived EVs, thereby minimizing interference from non-neuronal EVss with the experimental results. Third, the sample size was relatively small, partly because of disruptions in follow-up caused by the COVID-19 pandemic in 2019. Meanwhile, we did not adjust for several potential confounders such as diet, cardiovascular health, depression and socioeconomic status, and potential reverse causation cannot be fully excluded since individuals with better baseline health are more likely to engage in regular PA. Nonetheless, our findings provide important longitudinal evidence for the role of PA intensity in regulating synaptic function during aging and AD progression, and warrant validation in larger, more diverse populations to assess their generalizability to different demographic groups.

In summary, our study implied that synaptic biomarkers, including GAP43, Ng, SNAP25, and synaptotagmin 1, declined more rapidly in preclinical AD than in aging. Moreover, higher PA appears to slow the loss of these synaptic proteins, although its protective effects may differ with AD pathology. Collectively, these findings suggest that higher PA may regulate synaptic homeostasis through disease-dependent neuroprotective mechanisms and may offer intervention potential in the early stages of AD. Further studies with larger sample sizes and longer follow-up durations are required to validate these findings and explore the underlying biological mechanisms.

## Materials and methods

### Study design and participants

This 10-year longitudinal cohort study included 231 participants who were cognitively normal at baseline (preclinical AD, 115; controls, 116). Detailed recruitment, screening and exclusion procedures leading to this final sample are summarized in Fig. S9. All participants underwent comprehensive clinical assessments and concurrent collection of blood and CSF samples every two years. The preclinical AD group comprised individuals who possessed intact cognitive abilities at baseline but developed AD during the 10-year follow-up period. In contrast, the control group included participants who remained cognitively normal throughout the study period. The clinical diagnosis of AD was based on the criteria of the National Institute on Aging and the Alzheimer’s Association (2011) [49]. Based on our previous studies, P-tau181/Aβ42 > 0.14 and Aβ42 < 500 pg/mL in CSF were considered as additional criteria to distinguish between patients with AD and controls [50]. All participants originated from a longitudinal sub-cohort of the China Cognition and Aging Study (COAST) (*n* = 429). After matching on age, sex, and education level, eligible individuals with preclinical AD and controls were randomly selected using SPSS. This study was approved by the Ethics Committee of Xuanwu Hospital, Capital Medical University (ethics approval number [2023]151). Informed consent was obtained from all study participants or their legal guardians.

### Physical activity level assessment

We assessed participants’ PA levels using METs, a standardized unit used to quantify the energy expenditure of different PA intensities [13]. PA data were collected at each follow-up point using a self-reported health behavior questionnaire that recorded the type of activity, duration (in min), and frequency (times per week) over the past week [51] (Table S5). The MET intensity values for each activity were assigned according to the scoring protocol in the Compendium of Physical Activities [13]. The total weekly PA level for each participant was calculated by summing the MET minutes for all activity types. Participants were categorized into a higher PA group (≥ 500 MET-min/week) and a lower PA group (1–499 MET-min/week) according to previous studies and current guidelines [13, 52], and only participants who consistently met the predefined criteria at each follow-up time point were included in the final analysis.

#### Isolation of neuron-derived extracellular vesicles from the blood

Neuron-derived EVs were obtained and validated according to a previously published protocol [4]. Briefly, 20 mL whole blood samples were collected from all participants in EDTA-coated polypropylene tubes in the morning following a 12-h fast. The samples were immediately centrifuged at 4200 × *g* for 10 min at 4 °C to obtain plasma, which was then aliquoted and stored at −80 °C until neuron-derived EVs were isolated. The ExoQuick exosome precipitation solution (EXOQ; System Biosciences, USA) was used to collect total EVs from plasma, and co-immunoprecipitation was performed to isolate EVs derived from neurons using a mouse anti-human NCAM antibody. The EZ-Link sulfo-NHS-biotin system (Thermo Fisher Scientific, USA) was used to biotinylate EVs. Our previous study demonstrated that this procedure successfully enriched neuron-derived EVs [4, 53]. Additional details on EV isolation and characterization are provided in the Supplementary Materials (Fig. S10, Table S6).

#### CSF collection

CSF samples were collected, processed, and stored according to standard protocols [54]. Briefly, a lumbar puncture was performed by a trained physician to obtain CSF from the intervertebral space L3-L4 or L4-L5 using a 20-gauge needle. The collected CSF was centrifuged at 2000 × *g* for 10 min at room temperature, aliquoted, and stored in polypropylene tubes at −80 °C until further analysis.

#### Protein measurements

An Enzyme-Linked Immunosorbent Assay (ELISA) was performed to quantify the target proteins according to the manufacturer’s instructions. The target proteins included Aβ42, P-tau181, and T-tau in CSF, synaptic proteins GAP43, Ng, SNAP25, and synaptotagmin 1 in EVs, EV markers, and circulating exerkines BDNF, CTSB, IGF-1 and irisin in plasma. Details of the ELISA kits used in this study are described in Table S6 and in our previous study [4]. All procedures were performed in a blinded manner.

#### Cognitive function assessment

Global cognitive function was measured at baseline and each follow-up using the MMSE and MoCA. Both the MMSE and MoCA scores range from 0 to 30, with lower scores indicating poorer cognitive status [55, 56]. All cognitive assessments were administered by trained evaluators using standardized procedures in a blinded manner.

#### Statistical analysis

Demographic differences were assessed using analysis of variance (continuous variables) and contingency χ2 test (categorical variables). Linear mixed-effects models were constructed using the lme4 package in R to evaluate longitudinal changes in synaptic proteins across different MET levels or diagnostic groups. In these models, the four synaptic proteins were analyzed separately as dependent variables. Fixed effects included age, sex, *APOE ε4* carrier status, years of education, and time (visit). Participant ID was included as a random intercept to account for between-subject variability. The interaction between time and PA levels or diagnostic group (visit*group) was included to test whether longitudinal trajectories differed between groups. Bonferroni correction was applied for multiple comparisons if applicable. Additionally, t-tests were performed to assess group differences in synaptic protein levels by PA level and diagnostic status, after confirming normality, homogeneity of variance, and that these covariates were not significantly associated with synaptic protein levels. Linear regression models were further used to assess associations between average PA levels across all time points and synaptic protein levels at each follow-up visit, adjusting for the same covariates. Effect sizes were quantified using standardized beta coefficients (β) derived from the linear mixed-effects models. Statistical significance was defined as a two-tailed *P* < 0.05. All statistical analyses were performed using SPSS Statistics for Windows version 22.0 (IBM Corp., Armonk, NY, USA) and R software version 4.2.3 (R Foundation for Statistical Computing, Vienna, Austria).

## Supplementary Information

Supplementary Material 1.

## Data Availability

Data supporting the findings of this study are available upon request from the corresponding authors upon reasonable request.
