# Supplementary material for: Higher physical activity levels mitigate synaptic protein loss and cognitive deterioration in aging and in Alzheimer’s disease: a 10-year longitudinal study
Source: Mol Biomed. 2026 Jul 15;7:110. doi: 10.1186/s43556-026-00513-5 (PMC13373128; doi:10.1186/s43556-026-00513-5)
Supplement: Supplementary file 1 — Supplementary Material 1. [file 43556_2026_513_MOESM1_ESM.docx]

**Higher physical activity levels mitigate synaptic protein loss and cognitive deterioration in aging and in Alzheimer’s disease:** **a 10-year longitudinal study**

**Author information**

Shuiyue Quan, M.D.^1,2^; Xiaofeng Fu, M.D.^1,2^; Huimin Cai, M.D.^1,2^; Weiyun Zhang, M.D.^1,2^; Yumei Geng, M.D.^1,2^; Qing Tian, M.D.^1,2^; Ziye Ren, M.D.^1,2^; Yinghao Xu, M.D.^1,2^; Chengyu An, M.D.^1,2^; Jiaqi Li, M.D.^1,2^; Wei Wang, M.D. ^1,2^*; Longfei Jia, M.D., Ph.D^1,2^*

**Author Affiliations**

^1^Department of Neurology & Innovation Center for Neurological Disorders, Xuanwu Hospital, Capital Medical University, National Clinical Research Center for Geriatric Diseases, Beijing, 100053, China

^2^Beijing Key Lab of Clinical Translational Research in Cognitive, Affective and Behavioral Disorders, Xuanwu Hospital, Capital Medical University, Beijing, China

***Correspondence:** Longfei Jia.

Full address: Department of Neurology & Innovation Center for Neurological Disorders, Xuanwu Hospital, Capital Medical University, National Clinical Research Center for Geriatric Diseases, 45 Changchun St., Beijing, 100053, China. Tel.: +86 10 83199456; Fax: +86 10 63150669

E-mail: longfei@mail.ccmu.edu.cn; jialongfei@gmail.com.

***Correspondence:** Wei Wang.

Full address: Department of Neurology & Innovation Center for Neurological Disorders, Xuanwu Hospital, Capital Medical University, National Clinical Research Center for Geriatric Diseases, 45 Changchun St., Beijing, 100053, China. Tel.: +86 18201306651.

E-mail: sunnywangwei@163.com.

**Contents**

Fig.S1. Neuron-derived EV levels of synaptic proteins at the 10-year follow-up.

Fig.S2. Neuron-derived EV levels of synaptic proteins at baseline.

Fig.S3. Neuron-derived EV levels of synaptic proteins at baseline in patients with preclinical Alzheimer’s disease.

Fig.S4. Neuron-derived EV levels of synaptic proteins at baseline in controls.

Fig.S5. Correlations between physical activity and synaptic proteins in neuron-derived extracellular vesicles across follow-up in controls.

Fig.S6. Levels of plasma exerkines at the 10-year follow-up in controls.

Fig.S7. Correlations between physical activity and synaptic proteins in neuron-derived extracellular vesicles across follow-up in Alzheimer’s disease.

Fig.S8. Levels of plasma exerkines at the 10-year follow-up in patients with preclinical Alzheimer’s disease.

Fig.S9. Flowchart of study participants.

Fig.S10. Characterization and quality control of neuron-derived EVs.

Table S1. Longitudinal changes of synaptic protein levels in neuron-derived extracellular vesicles during aging and Alzheimer’s disease progression.

Table S2. β coefficients and 95% confidence intervals for longitudinal changes of synaptic proteins and cognitive function.

Table S3. Longitudinal changes of synaptic protein levels in neuron-derived extracellular vesicles stratified by MET levels during aging.

Table S4. Longitudinal changes of synaptic protein levels in neuron-derived extracellular vesicles stratified by MET levels during Alzheimer’s disease progression.

Table S5. Physical Activity Questionnaire.

Table S6. ELISA kits information.

**
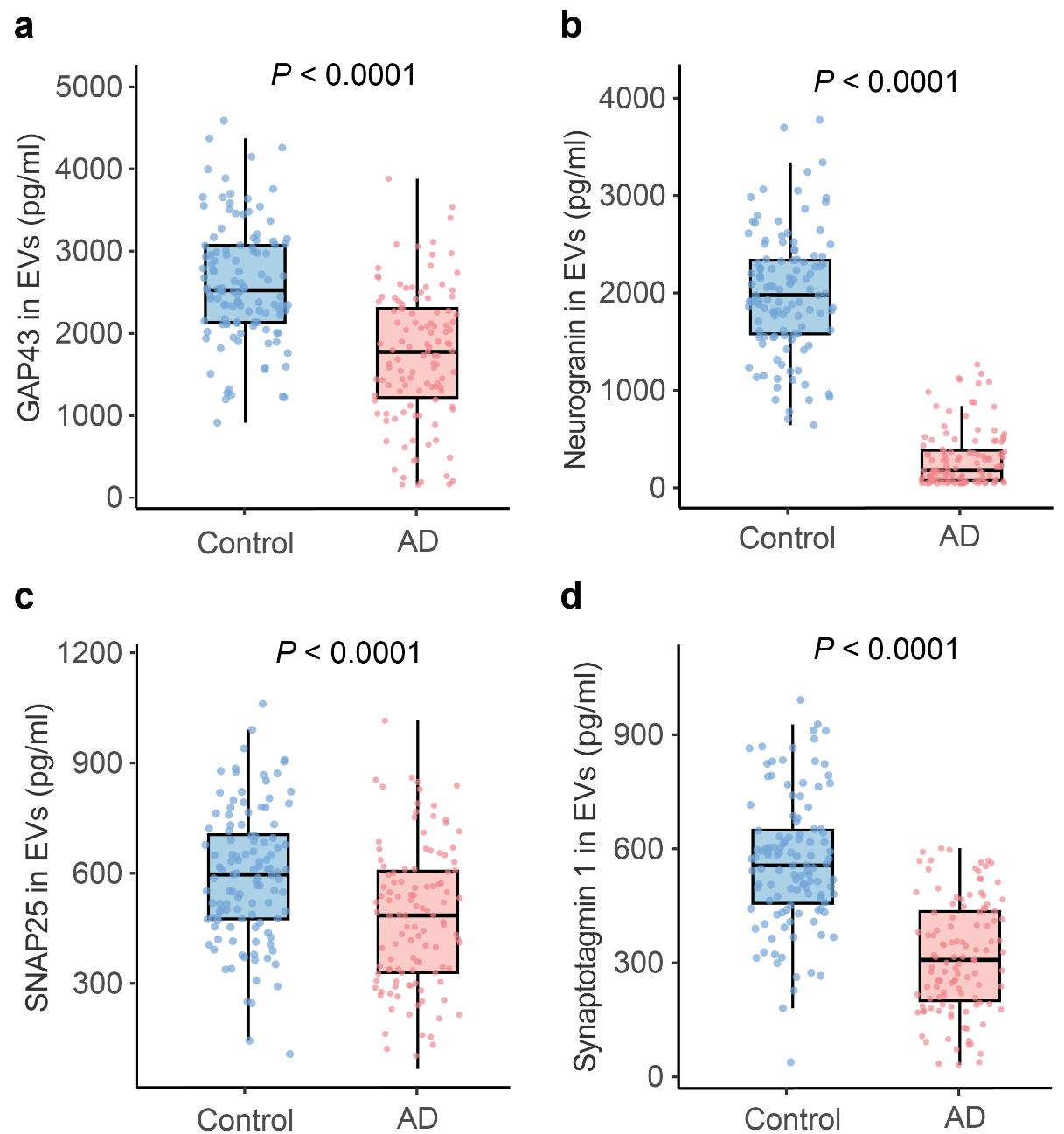
**

**Fig.S1. Neuron-derived EV levels of synaptic proteins at the 10-year follow-up.** Neuron-derived EV levels of growth-associated protein 43 (GAP43) (**a**), neurogranin (**b**), synaptosomal-associated protein 25 (SNAP25) (**c**), and synaptotagmin 1 (**d**) were measured at the 10-year follow-up. The *P*-values from t-tests comparing participants in the AD and control groups are shown in each panel. *n* = 116 (control); *n* = 115 (AD). EV, extracellular vesicle; AD, Alzheimer’s disease.

**
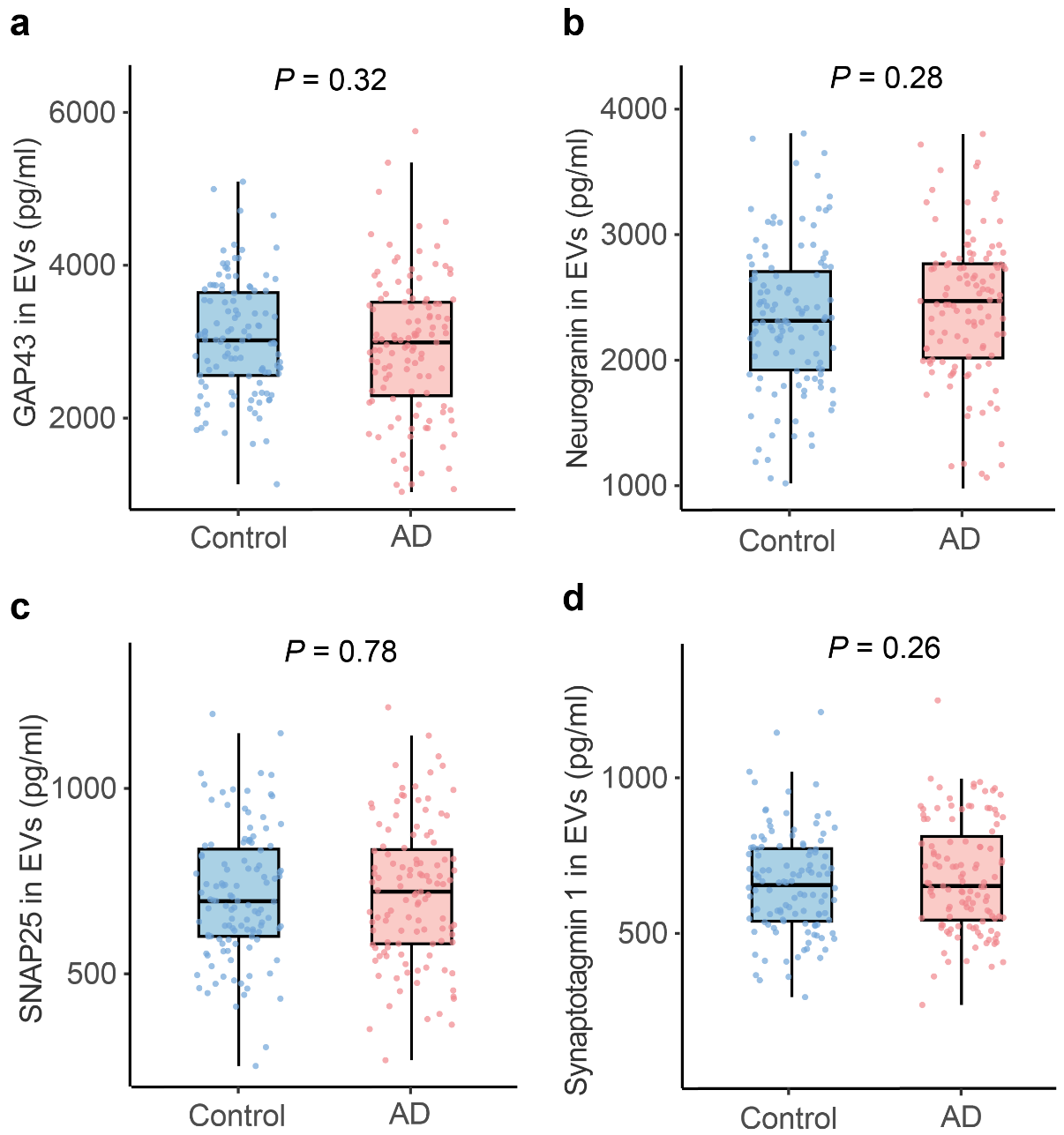
**

**Fig.S2. Neuron-derived EV levels of synaptic proteins at baseline.** Neuron-derived EV levels of growth-associated protein 43 (GAP43) (**a**), neurogranin (**b**), synaptosomal-associated protein 25 (SNAP25) (**c**), and synaptotagmin 1 (**d**) were measured at baseline. The *P*-values from t-tests comparing participants in the preclinical AD and control groups are shown in each panel. *n* = 116 (control); *n* = 115 (AD). EV, extracellular vesicle; AD, Alzheimer’s disease.

**
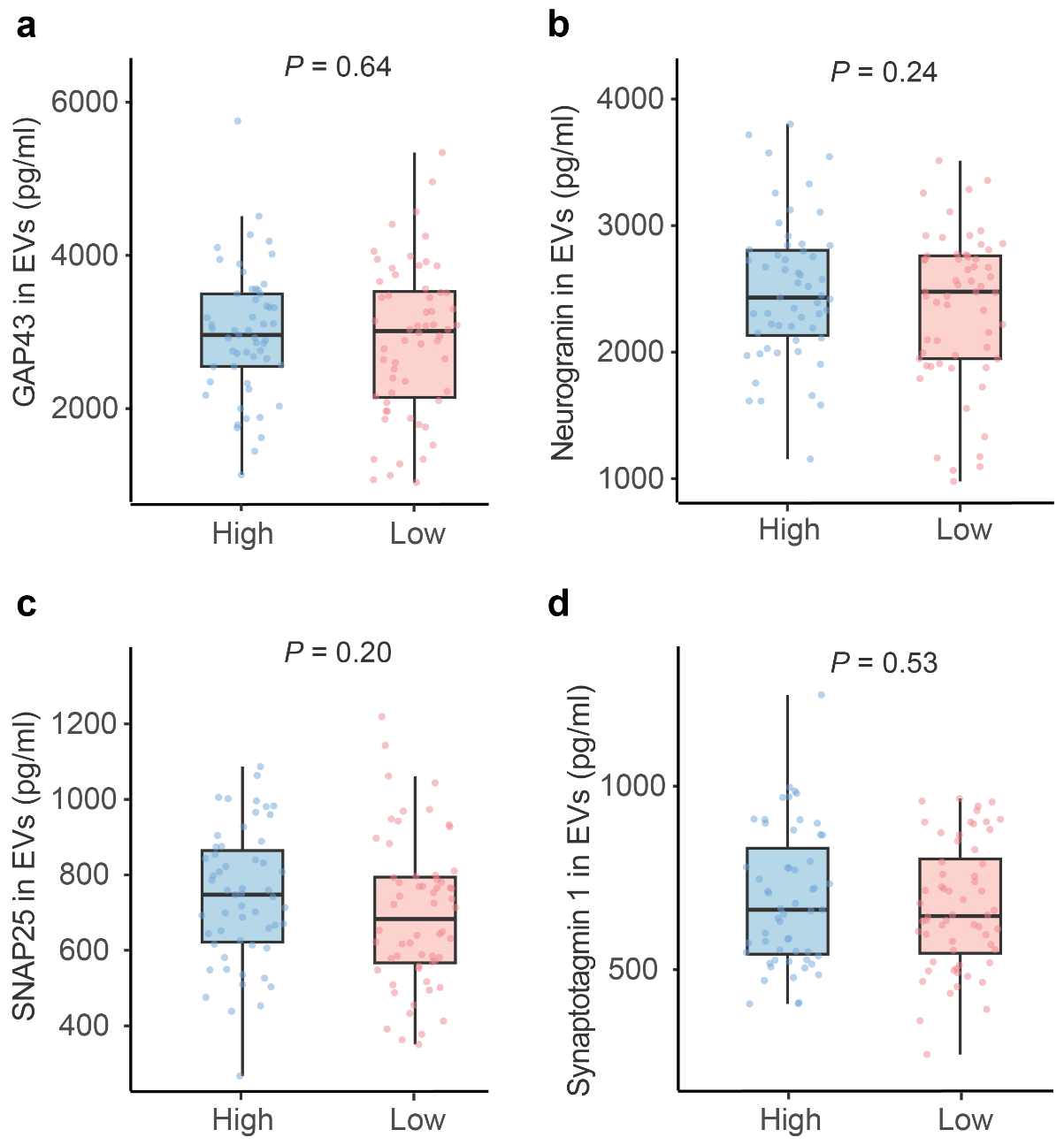
**

**Fig.S3. Neuron-derived EV levels of synaptic proteins at baseline in patients with preclinical Alzheimer’s disease.** Neuron-derived EV levels of growth-associated protein 43 (GAP43) (**a**), neurogranin (**b**), synaptosomal-associated protein 25 (SNAP25) (**c**), and synaptotagmin 1 (**d**) were measured at baseline in patients with preclinical AD. The *P*-values from the t-tests comparing participants in the high- and low-MET groups are shown in each panel. *n* = 60 (low METs) and *n* = 55 (high METs). EV, extracellular vesicle; MET, metabolic equivalent threshold; AD, Alzheimer’s disease**.**

**
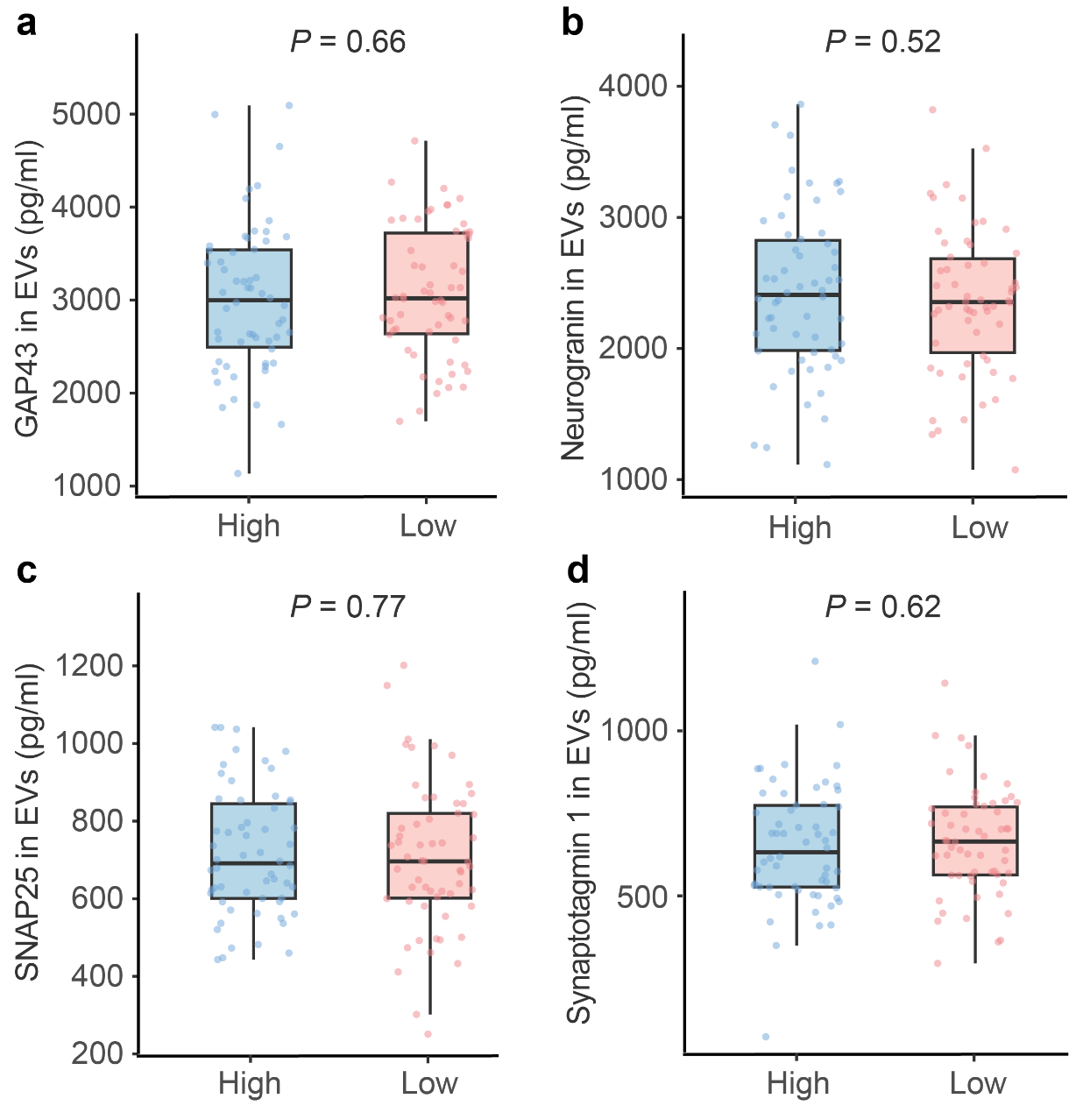
**

**Fig.S4. Neuron-derived EV levels of synaptic proteins at baseline in controls.** Neuron-derived EV levels of growth-associated protein 43 (GAP43) (**a**), neurogranin (**b**), synaptosomal-associated protein 25 (SNAP25) (**c**), and synaptotagmin 1 (**d**) were measured at baseline in controls with low and high MET levels. The *P*-values from the t-tests comparing participants in the high- and low- METs groups are shown in each panel. *n* = 58 (low METs); *n* = 58 (high METs). EV, extracellular vesicle; MET, metabolic equivalent threshold**.**

**
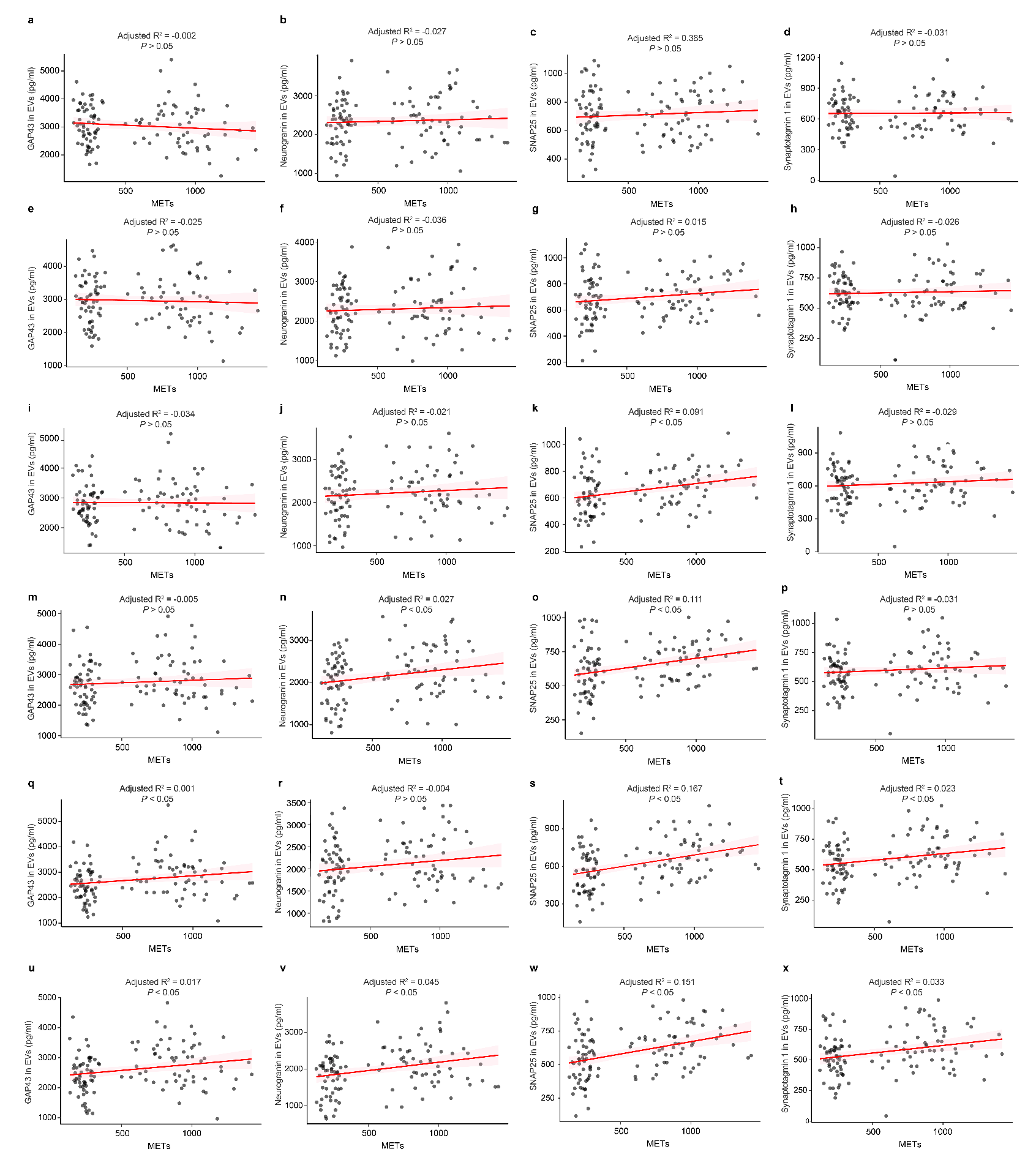
**

**Fig.S5. Correlations between physical activity and synaptic proteins in neuron-derived extracellular vesicles across follow-up in controls.** Panels (**a-d**), (**e-h**), (**i-l**), (**m-p**), (**q-t)**, and (**u-x**) show the associations between physical activity and synaptic protein levels at baseline, 2-, 4-, 6-, 8-, and 10-year follow-up, respectively. Solid lines indicate the correlation. *n* = 116 (controls).


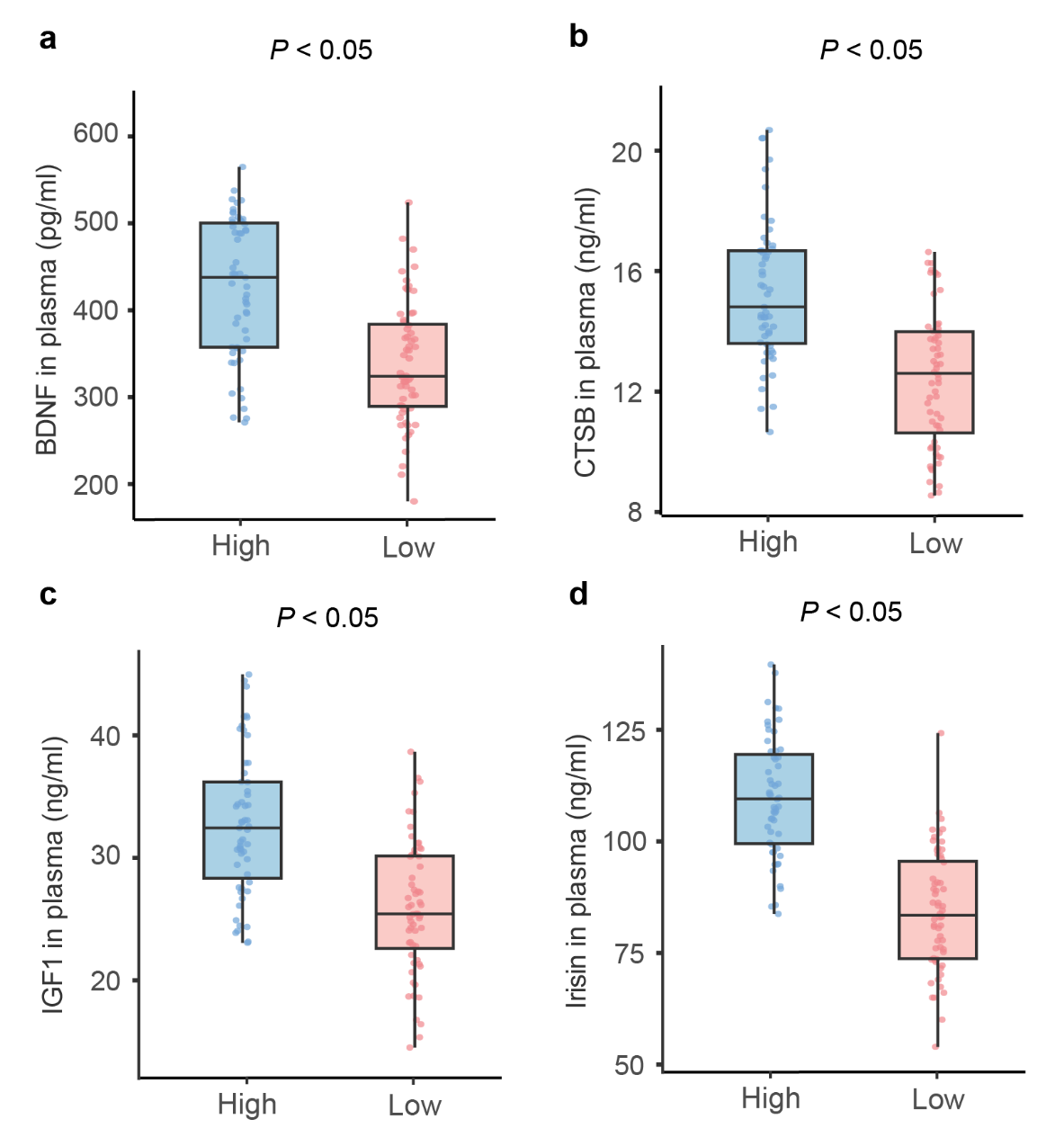


**Fig.S6. Levels of plasma exerkines at the 10-year follow-up in controls.** Levels of brain-derived neurotrophic factor (BDNF) (**a**), cathepsin B (CTSB) (**b**), insulin-like growth factor 1 (IGF1) (**c**), and irisin (**d**) were measured at the 10-year follow-up in controls with low and high MET levels. The *P*-values from the t-tests comparing participants in the high- and low-MET groups are shown in each panel. *n* = 58 (low METs) and *n* = 58 (high METs)**.**

**
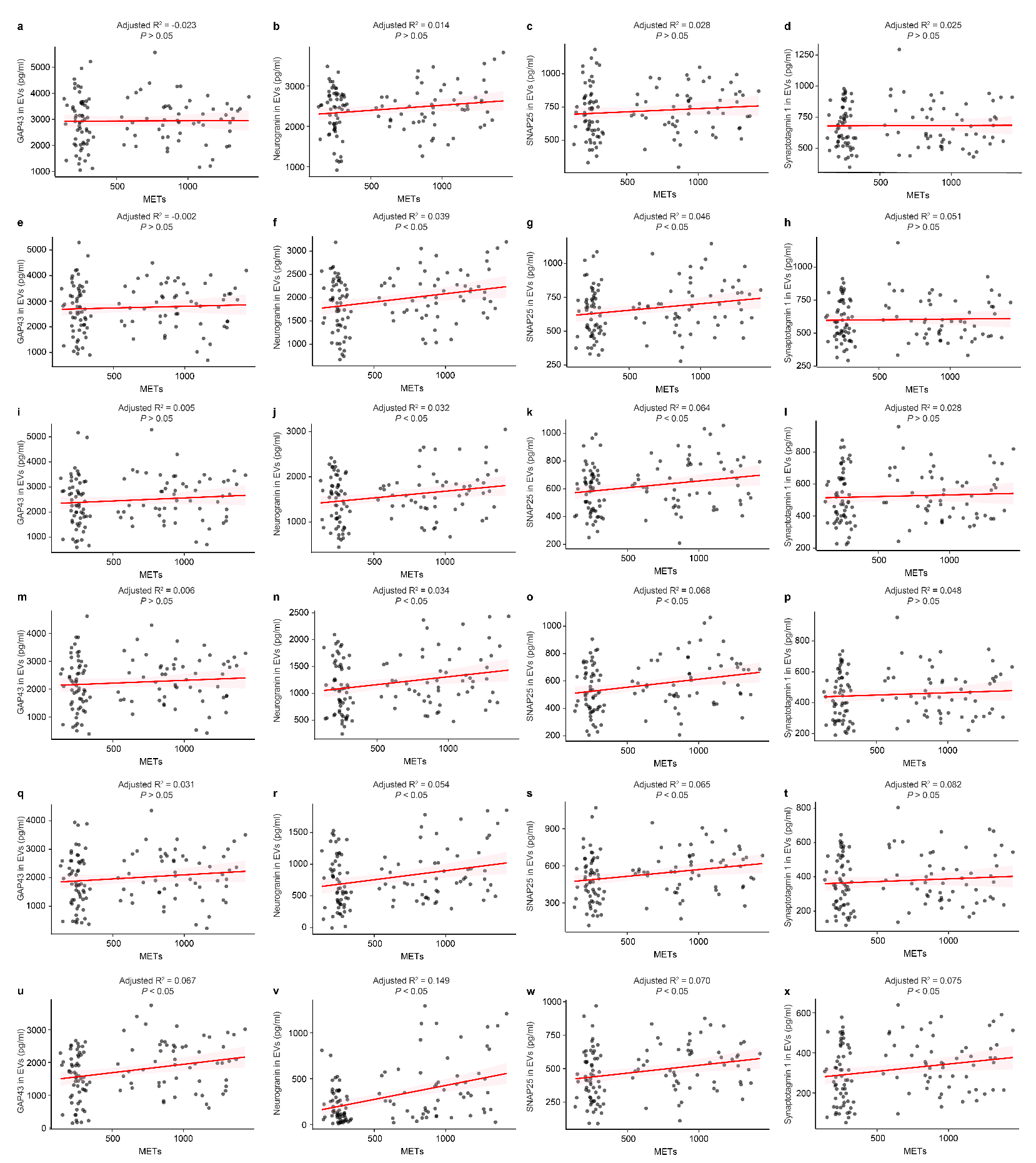
**

**Fig.S7. Correlations between physical activity and synaptic proteins in neuron-derived extracellular vesicles across follow-up in Alzheimer’s disease.** Panels (**a-d**), (**e-h**), (**i-l**), (**m-p**), (**q-t)**, and (**u-x**) show the associations between physical activity and synaptic protein levels at baseline, 2-, 4-, 6-, 8-, and 10-year follow-up, respectively. Solid lines indicate the correlation. *n* = 115 (Alzheimer’s disease).


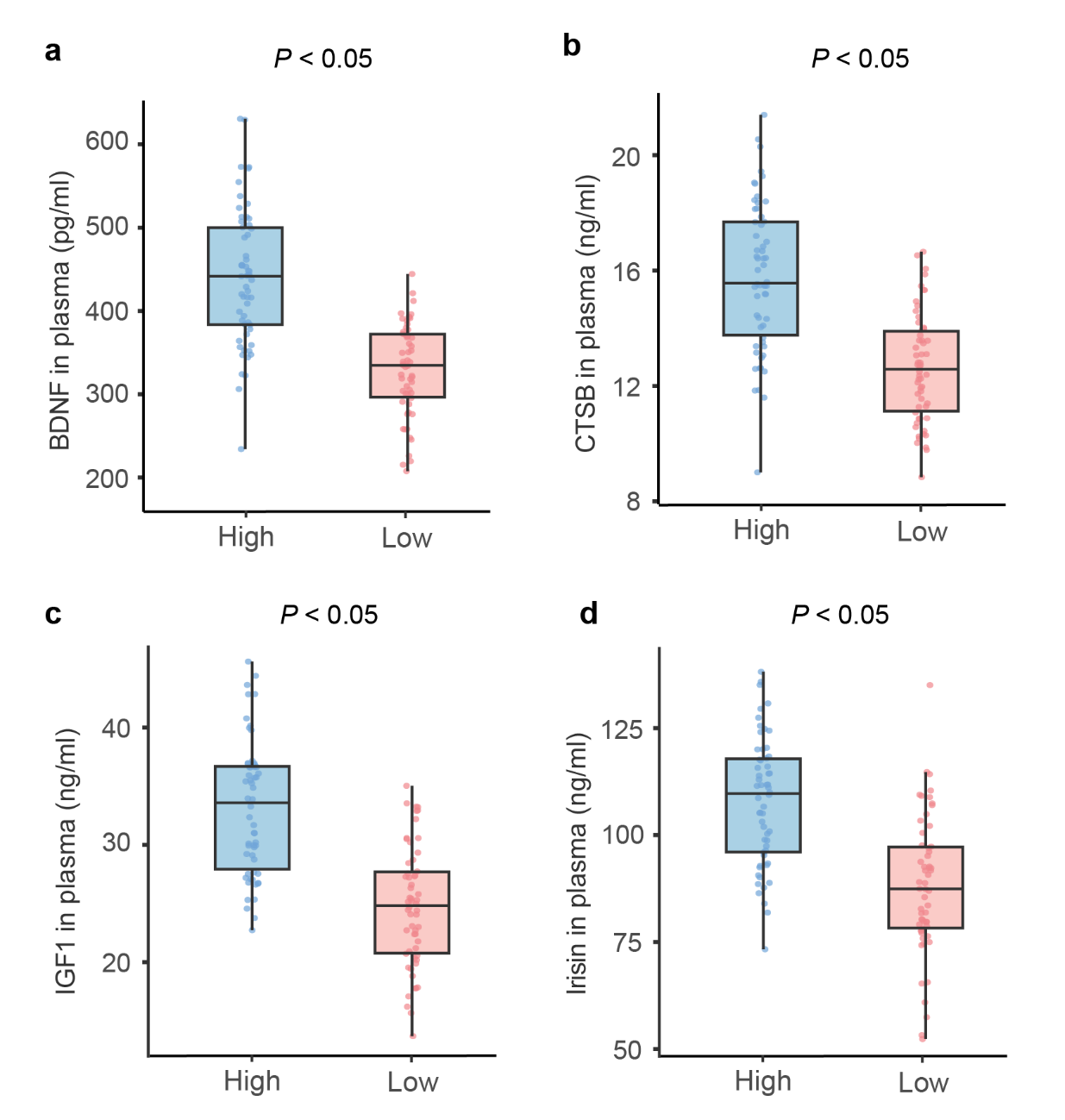


**Fig.S8. Levels of plasma exerkines at the 10-year follow-up in patients with preclinical Alzheimer’s disease.** Levels of brain-derived neurotrophic factor (BDNF) (**a**), cathepsin B (CTSB) (**b**), insulin-like growth factor 1 (IGF1) (**c**), and irisin (**d**) were measured at the 10-year follow-up in patients with preclinical Alzheimer’s disease with low and high MET levels. The *P*-values from the t-tests comparing participants in the high- and low-MET groups are shown in each panel. *n* = 60 (low METs) and *n* = 55 (high METs)**.**


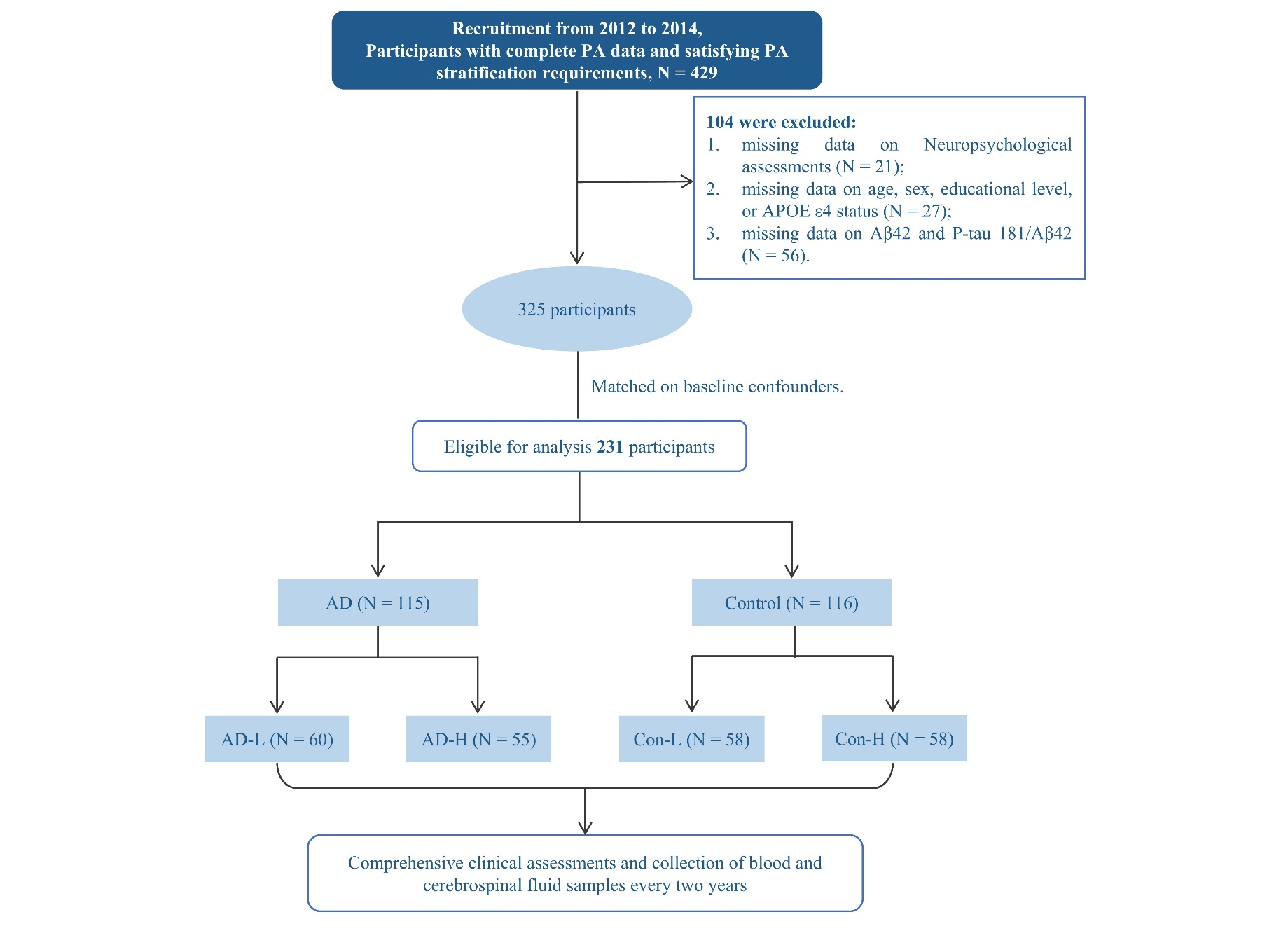


**Fig.S9. Flowchart of study participants.**

**
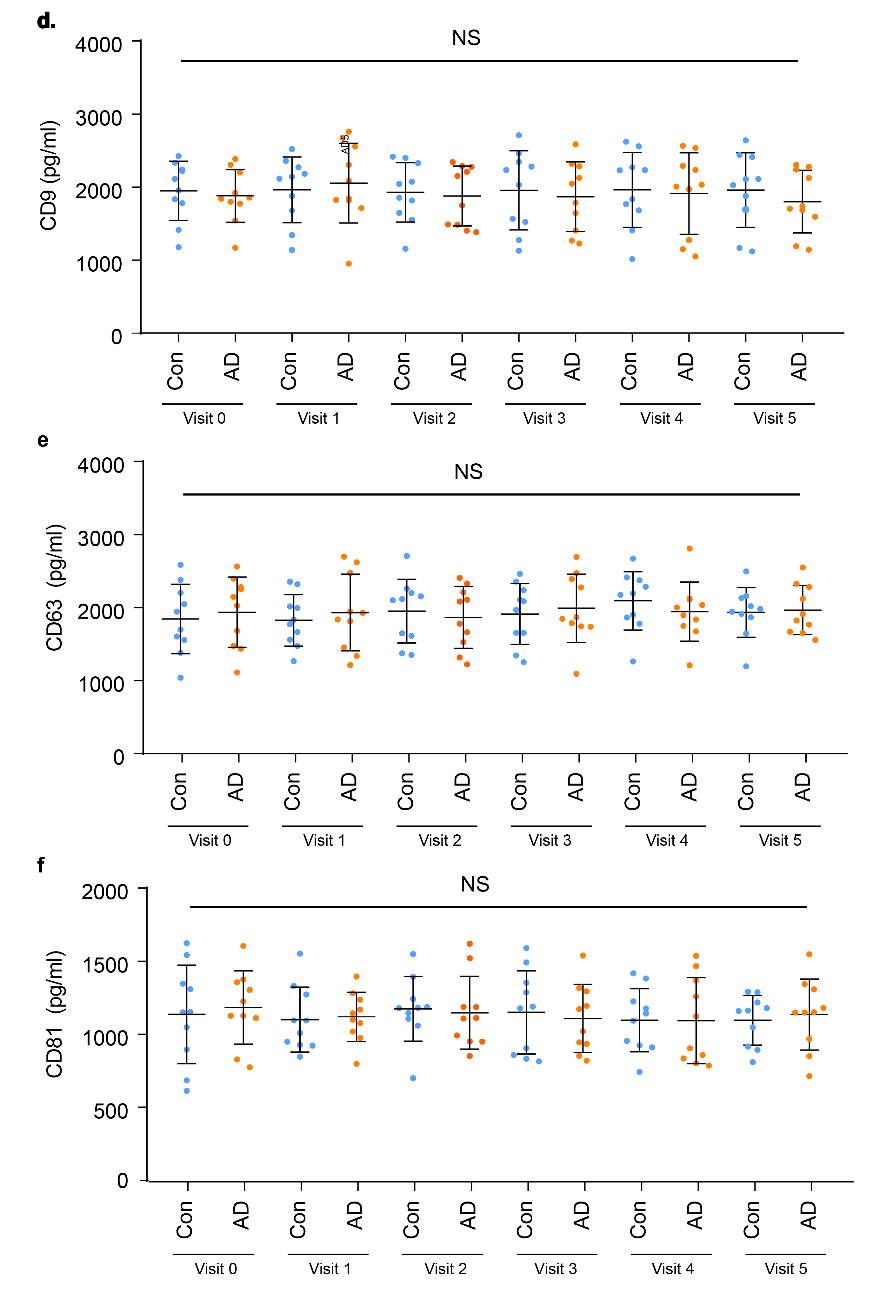

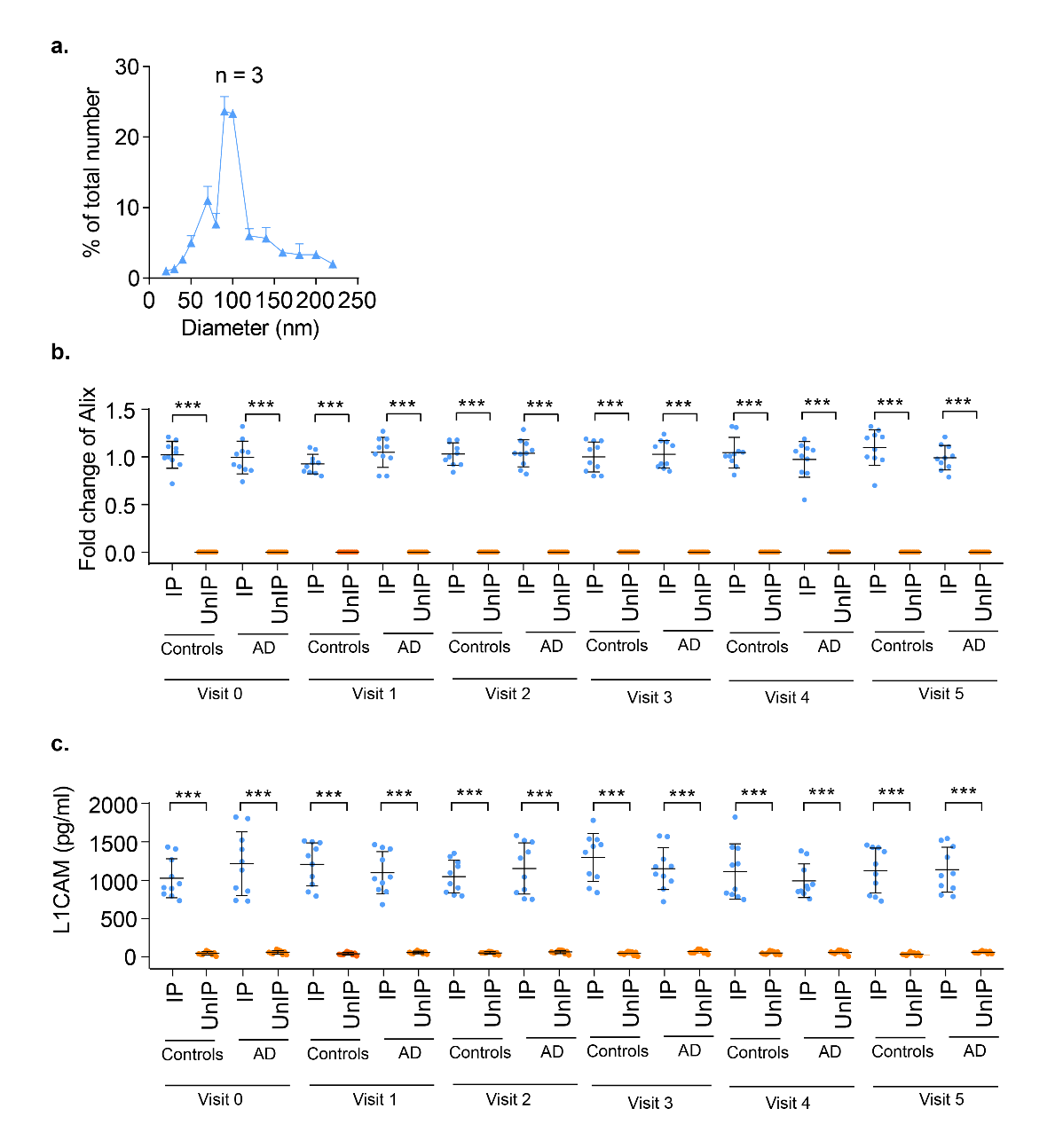
**

**Fig.S10. Characterization and quality control of neuron-derived EVs.** (**a**) Particle size distribution of plasma neuron-derived EVs assessed by NTA. (**b**) Validation of EV enrichment by immunoprecipitation. Alix levels were significantly enriched in the immunoprecipitated (IP) fraction compared with the unprecipitated (UnIP) fraction across all visits and diagnostic groups. (**c**) Enrichment of neuronal-associated marker L1CAM in the IP fraction compared with the UnIP fraction across all visits and diagnostic groups. (**d-f**) Levels of canonical EV surface markers CD9 (**d**), CD63 (**e**), and CD81 (**f**) measured across visits in controls and AD groups. NTA, nanoparticle tracking analysis；L1CAM, L1 cell adhesion molecule.

**Table S1. Longitudinal changes of synaptic protein levels in neuron-derived extracellular vesicles during aging and Alzheimer’s disease progression**

| Synaptic markers |  | AD | |  |  | Control | |  |
| --- | --- | --- | --- | --- | --- | --- | --- | --- |
|  | β estimate | | *P-*value | | β estimate | | *P-*value | |
| GAP43 | -241.43 | | < 0.001 | | -85.14 | | < 0.001 | |
| Ng | -418.68 | | < 0.001 | | -77.04 | | < 0.001 | |
| SNAP25 | -47.81 | | < 0.001 | | -24.19 | | < 0.001 | |
| Synaptotagmin 1 | -79.03 | | < 0.001 | | -22.87 | | < 0.001 | |

*Note:* The β-estimates and *P-*values are derived from linear mixed effects models with the interaction between time from baseline and synaptic marker slopes after adjusting for age, sex, *APOE* ε4 carrier status, and years of education. *n* = 115 (AD), *n* = 116 (Control).

Abbreviations: AD, Alzheimer’s disease; GAP43, growth-associated protein 43, Ng, neurogranin; SNAP25, synaptosomal-associated protein 25.

**Table S2. β coefficients and 95% confidence intervals for longitudinal changes of synaptic proteins and cognitive function**

| **Marker & cognitive function** | **β estimate** | **95% CIs** | ***P*-value** |
| --- | --- | --- | --- |
| **Fig.2 (Control)** |  |  |  |
| GAP43 | -81.32 | (-289.69, 127.05) | 0.445 |
| Ng | 45.87 | (-93.32, 185.06) | 0.519 |
| SNAP25 | 1.13 | (-44.89, 47.15) | 0.962 |
| Synaptotagmin 1 | 27.60 | (-14.10, 69.31) | 0.196 |
| **Fig.2 (AD)** |  |  |  |
| GAP43 | -155.04 | (-171.39, -138.70) | < 0.001 |
| Ng | -342.57 | (-355.04, -330.09) | < 0.001 |
| SNAP25 | -23.64 | (-27.18, -20.09) | < 0.001 |
| Synaptotagmin 1 | -56.56 | (-59.71, -53.40) | < 0.001 |
| **Fig.5 (Con-H)** |  |  |  |
| GAP43 | -55.93 | (-96.14, -15.72) | 0.007 |
| Ng | -52.85 | (-86.22, -19.48) | 0.002 |
| SNAP25 | -8.59 | (-17.90, 0.72) | 0.073 |
| Synaptotagmin 1 | -10.22 | (-19.60, -0.84) | 0.034 |
| **Fig.5 (Con-L)** |  |  |  |
| GAP43 | -161.82 | (-202.41, -121.23) | < 0.001 |
| Ng | -116.45 | (-150.08, -82.81) | < 0.001 |
| SNAP25 | -34.44 | (-43.84, -25.05) | < 0.001 |
| Synaptotagmin 1 | -32.07 | (-41.52, -22.62) | < 0.001 |
| **Fig.6 (AD-H)** |  |  |  |
| GAP43 | -181.21 | (-231.05, -131.38) | < 0.001 |
| Ng | -407.02 | (-435.35, -378.69) | < 0.001 |
| SNAP25 | -43.50 | (53.80, -33.18) | < 0.001 |
| Synaptotagmin 1 | -75.50 | (-84.22, -66.78) | < 0.001 |
| **Fig.6 (AD-L)** |  |  |  |
| GAP43 | -250.01 | (-299.26, -200.77) | < 0.001 |
| Ng | -418.93 | (-446.60, -391.27) | < 0.001 |
| SNAP25 | -55.74 | (-65.92, -45.55) | < 0.001 |
| Synaptotagmin 1 | -84.94 | (-93.56, -76.33) | < 0.001 |
| **Fig.7 (AD-H)** |  |  |  |
| MMSE | -1.73 | (-1.84, -1.63) | < 0.001 |
| MoCA | -2.75 | (-2.86, -2.64) | < 0.001 |
| **Fig.7 (AD-L)** |  |  |  |
| MMSE | -2.10 | (-2.20, -2.00) | < 0.001 |
| MoCA | -3.07 | (-3.17, -2.97) | < 0.001 |

*Note:* The β-estimates, 95% confidence intervals (95% CIs) and *P*-values are derived from linear mixed effects models, corresponding to Figure 2, Figure 5, Figure 6 and Figure 7. *n* = 115 (AD), *n* = 116 (Control); *n* = 58 (Con-L), *n* = 58 (Con-H); *n* = 60 (AD-L), *n* = 55 (AD-H).

Abbreviations: METs, metabolic equivalents; Con-L, lower METs levels in control; Con-H, higher METs levels in control; AD, Alzheimer’s disease; AD-L, lower METs levels in AD; AD-H, higher METs levels in AD; GAP43, growth-associated protein 43, Ng, neurogranin; SNAP25, synaptosomal-associated protein 25; MMSE, Mini-Mental State Examination; MoCA, Montreal Cognitive Assessment.

**Table S3. Longitudinal changes of synaptic protein levels in neuron-derived extracellular vesicles stratified by MET levels during aging**

| Synaptic markers |  | Con-L | |  |  | Con-H | |  |
| --- | --- | --- | --- | --- | --- | --- | --- | --- |
|  | β estimate | | *P-*value | | β estimate | | *P-*value | |
| GAP43 | -161.36 | | < 0.001 | | -55.48 | | 0.008 | |
| Ng | -116.38 | | < 0.001 | | -52.78 | | 0.002 | |
| SNAP25 | -34.67 | | < 0.001 | | -8.84 | | 0.070 | |
| Synaptotagmin 1 | -32.09 | | < 0.001 | | -10.24 | | 0.033 | |

*Note:* The β-estimates and *P-*values are derived from linear mixed effects models with the interaction between time from baseline and synaptic marker slopes after adjusting for age, sex, *APOE* ε4 carrier status, and years of education. *n* = 58 (Con-L), *n* = 58 (Con-H).

Abbreviations: METs, metabolic equivalents; Con-L, lower METs levels in control; Con-H, higher METs levels in control; GAP43, growth-associated protein 43, Ng, neurogranin; SNAP25, synaptosomal-associated protein 25.

**Table S4. Longitudinal changes in synaptic protein levels in neuron-derived extracellular vesicles stratified by MET levels during Alzheimer’s disease progression**

| Synaptic markers |  | AD-L | |  |  | AD-H | |  |
| --- | --- | --- | --- | --- | --- | --- | --- | --- |
|  | β estimate | | *P-*value | | β estimate | | *P-*value | |
| GAP43 | -253.09 | | < 0.001 | | -184.32 | | < 0.001 | |
| Ng | -420.54 | | < 0.001 | | -408.69 | | < 0.001 | |
| SNAP25 | -56.20 | | < 0.001 | | -43.96 | | < 0.001 | |
| Synaptotagmin 1 | -85.93 | | < 0.001 | | -76.51 | | < 0.001 | |
| MMSE | -2.10 | | < 0.001 | | -1.74 | | < 0.001 | |

*Note:* The β-estimates and *P*-values are derived from linear mixed effects models with the interaction between time from baseline and synaptic proteins or MMSE scores, adjusted for age, sex, *APOE* ε4 carrier status, and years of education. *n* = 60 (AD-L), *n* = 55 (AD-H)

Abbreviations: METs, metabolic equivalents; AD, Alzheimer’s disease; AD-L, lower METs levels in AD; AD-H, higher METs levels in AD; GAP43, growth-associated protein 43, Ng, neurogranin; SNAP25, synaptosomal-associated protein 25; MMSE, Mini-Mental State Examination.

| **Table S5. Physical Activity Questionnaire** | |
| --- | --- |
| **Part I. Self-reported by participants** | |
| These questions asked you about the time you spent being physically active in the past year. | |
| 1. Did you walk more than 10,000 steps each time? | Yes ☐ No☐ |
| If yes, how many times per week? | ≥7 times per week ☐  ≤ 6 times per week ☐  ≤ 5 times per week ☐  ≤ 4 times per week ☐  ≤ 3 times per week ☐  ≤ 2 times per week ☐  ≤ 1 time per week≤ ☐ |
| 2. Did you often take Tai Chi exercise (exceeding 30 minutes each time)? | Yes ☐ No☐ |
| If yes, how many times per week? | ≥7 times per week ☐  ≤ 6 times per week ☐  ≤ 5 times per week ☐  ≤ 4 times per week ☐  ≤ 3 times per week ☐  ≤ 2 times per week ☐  ≤ 1 time per week ☐ |
| 3. Did you regularly participate in moderate-intensity physical exercise (requiring moderate effort and accelerating the heart rate)? | Yes☐ No☐ |
| If yes, please specify the type(s) of activity?  _____________________  How many times per week? | ≥7 times per week ☐  ≤ 6 times per week ☐  ≤ 5 times per week ☐  ≤ 4 times per week ☐  ≤ 3 times per week ☐  ≤ 2 times per week ☐  ≤ 1 time per week ☐ |
| How long in total per week? | ___minutes per week |
| 4. Did you regularly participate in vigorous activity (requires a large amount of effort and causes rapid breathing and a substantial increase in heart rate), such as running, fast swimming, etc.? | Yes☐ No☐ |
| If yes, please specify the type(s) of activity?  _____________________  How many times per week? | ≥7 times per week ☐  ≤ 6 times per week ☐  ≤ 5 times per week ☐  ≤ 4 times per week ☐  ≤ 3 times per week ☐  ≤ 2 times per week ☐  ≤ 1 time per week ☐ |
| How long in total per week? | ___minutes per week |
| 5. Do you participate in any other types of physical activity that are not listed above? |  |
| If yes, please specify the type(s) of activity?  _____________________ |  |
| How many times per week? | ≥7 times per week ☐  ≤ 6 times per week ☐  ≤ 5 times per week ☐  ≤ 4 times per week ☐  ≤ 3 times per week ☐  ≤ 2 times per week ☐  ≤ 1 time per week ☐ |
| How long in total per week? | ___minutes per week |
| **Part II. Reviewed by investigators** | |
| Please recheck and evaluate the frequency and time spent on the following activities | |
| **Whether participants have the following physical exercise habits** | **Answers** |
| Walk more than 10000 steps | ≥7 times per week ☐  6 times per week ☐  5 times per week ☐  4 times per week ☐  3 times per week ☐  2 times per week ☐  1 time per week ☐  Never ☐  ___minutes per week |
| Take Tai Chi exercise (exceeding 30 minutes each time) | ≥7 times per week ☐  ≤ 6 times per week ☐  ≤ 5 times per week ☐  ≤ 4 times per week ☐  ≤ 3 times per week ☐  ≤ 2 times per week ☐  ≤ 1 time per week ☐  ≤ Never ☐  ___minutes per week |
| Regularly participate in moderate-intensity physical exercise | ≥7 times per week ☐  ≤ 6 times per week ☐  ≤ 5 times per week ☐  ≤ 4 times per week ☐  ≤ 3 times per week ☐  ≤ 2 times per week ☐  ≤ 1 time per week ☐  ≤ Never ☐  __minutes per week |
| Regularly participate in vigorous activity | ≥7 times per week ☐  ≤ 6 times per week ☐  ≤ 5 times per week ☐  ≤ 4 times per week ☐  ≤ 3 times per week ☐  ≤ 2 times per week ☐  ≤ 1 time per week ☐  ≤ Never ☐  __minutes per week |
| Other types of physical activity that are not listed above | ≥7 times per week ☐  ≤ 6 times per week ☐  ≤ 5 times per week ☐  ≤ 4 times per week ☐  ≤ 3 times per week ☐  ≤ 2 times per week ☐  ≤ 1 time per week ☐  ≤ Never ☐  __minutes per week |
| Physical exercise: __ |  |

**Table S6. ELISA kits information**

| **ELISA kits Catalog numbers** | | **ELISA kits Catalog numbers** | |
| --- | --- | --- | --- |
| **GAP43** | |  | |
| MyBiosource (USA） | | MBS2502209 | |
| **Neurogranin** | |  | |
| American Research Products (USA) | | CEA404Hu | |
| **SNAP25** | |  | |
| Proteintech (USA) | | KE00031 | |
| **Synaptotagmin 1** | |  | |
| Abbkine (China) | | KTE60424 | |
| **L1CAM** | |  | |
| LifeSpan BioSciences (USA) | | LS-F24209-1 | |
| **ALIX** | |  | |
| Abcam (UK) | | ab289653 | |
| **CD9** | |  | |
| LifeSpan BioSciences (USA) | | LS-F6853 | |
| **CD63** | |  | |
| RayBiotech (USA) | | ELH-CD63-1 | |
| **CD81** | |  | |
| LifeSpan BioSciences (USA) | | LS-F7468-1 | |
| **Aβ42** | |  | |
| INNOTEST (Japan） | | 81576 | |
| **T-tau** | |  | |
| INNOTEST (Japan） | | 81572 | |
| **P-T181-tau** | |  | |
| INNOTEST (Japan) | | 81581 | |
| **Cathepsin B** | |  | |
| Abcam | | AB119584 | |
| **BDNF** | |  | |
| R&D Systems | | DBD00 | |
| **IGF-1** | |  | |
| R&D Systems | | DG100 | |
| **Irisin** | |  | |
| Cusabio | | CSB-EQ027943HU | |
